# Automated wave runup monitoring using coastal CCTV cameras for tsunami detection

**DOI:** 10.1038/s41598-025-28874-x

**Published:** 2025-11-29

**Authors:** Tomoki Shirai, Taro Arikawa

**Affiliations:** 1 Research Fellow of Japan Society for the Promotion of Science (JSPS), Graduate School of Science and Engineering, Chuo University, 1-13-27 Kasuga, Bunkyo-Ku, Tokyo, 112-8551 Japan; 2 Department of Civil and Environmental Engineering, Chuo University, 1-13-27 Kasuga, Bunkyo-Ku, Tokyo, 112-8551 Japan

**Keywords:** Tsunami, Detection, Wave runup, CCTV, 2024 Noto Peninsula earthquake, Civil engineering, Natural hazards

## Abstract

Coastal closed-circuit television (CCTV) cameras are ubiquitous yet rarely exploited quantitatively for tsunami detection. To address this gap and the scarcity of automated methods, we propose a technique that converts CCTV footage into a time-series of wave runup heights—instantaneous shoreline elevations corresponding to each incoming wave. The workflow has two main steps: (i) compute a luminance-variation (SIGMA) image in which the runup edge appears as a bright curve, and (ii) apply a color-based land–water mask to suppress dynamic noise on land. Preliminary tests under varied lighting, wave, and obstacle conditions confirmed the method’s stability. Application to footage from seven CCTV cameras during the 1 January 2024 Mw 7.5 Noto Peninsula tsunami revealed one of the tsunami’s dominant 300–500 s energy band and yielded root-mean-square errors of 0.094 m and 0.191 m at two sites after removing short-period (< 180 s) components—while processing ran faster than real time on a standard computer. In the cases studied, the extracted runup time-series demonstrated the potential to complement sparse offshore gauges for real-time tsunami detection and post-event analysis. Future work will target a broader range of recording conditions, refine signal separation, and validate the method on additional tsunami events with characteristics different from the Noto case.

**Supplementary Information:**

The online version contains supplementary material available at 10.1038/s41598-025-28874-x.

## Introduction

Tsunami early warning systems primary depend on offshore observation networks^1^. These networks, however, remain sparse and expensive to deploy worldwide [2], limiting real-time detection capability in many coastal regions.

In coastal engineering, closed-circuit television (CCTV) cameras offer a cost-effective means of monitoring sea-surface variations, including tsunami-induced changes. CCTV footage has long been used to study nearshore processes—wave runup and shoreline movement^3–5^ and surf-zone breaker heights^6^. More recently, researchers have extended onshore CCTV monitoring seaward, using it to estimate offshore wave spectra^7^ and to apply stereo-matching for direct wave-height measurements^8,9^. These offshore-focused methods, however, are sensitive to environmental conditions (lighting, weather, and sea surface state) and require intensive processing of high-resolution imagery to resolve distant waters, keeping them at a research stage. Moreover, validating these offshore-focused algorithms presents a significant operational challenge. Since validation requires comparison against ground-truth data from dedicated offshore instruments, the inability to predict a tsunami’s time and location of origin makes it impractical to pre-deploy and maintain the necessary observation networks. This difficulty in performing in-situ validation raises reliability concerns for their operational use.

Here we focus on wave runup, described as the oscillatory movement of the shoreline relative to still‐water level, comprising a wave setup and swash fluctuations [10]. Runup observation benefits from well-established tsunami runup theory (e.g.,^11,12^), allowing straightforward physical interpretation of extracted runup time series. Additionally, compared with offshore-focused methods, focusing on shoreline displacement typically reduces sensitivity to illumination changes and allows monitoring with lower-resolution cameras, suggesting that it could be one of the most practical approaches for CCTV-based tsunami detection. A recognized limitation of coastal detection is the shorter local lead time compared to offshore systems. Nevertheless, it plays a crucial role as a complementary component to existing sparse offshore monitoring networks, particularly for detecting tsunamis that begin with a drawback or have a small initial wave, and for providing timely warnings to subsequent coastlines^13^.

More broadly, coastal video footage has long been utilized as a valuable data source for understanding tsunami behavior, spatially complementing sparse offshore observation networks. For instance, through post-event analysis of resident-filmed footage, Fritz et al.^14,15^ estimated inundation characteristics for the 2004 Indian Ocean and 2011 Great East Japan tsunamis. Similarly, Carvajal et al.^16^ analyzed videos from the 2018 Palu Tsunami, highlighting the crucial role of CCTV. More recently, the utility of video has been proven for smaller, non-inundating tsunamis, often through methods that rely on existing objects for scaling. A key example is McGill et al.^17^, who analyzed footage of the 2022 Tonga tsunami by using features like vessels to establish a vertical scale.

While valuable, these case-specific and often manual approaches differ from the goal of developing a more generalized, automated system. The recent tsunami triggered by the January 1, 2024 Noto Peninsula earthquake, recorded by numerous CCTV cameras^18–25^, provides an ideal dataset to develop and test such an automated approach. Shirai et al.^22^ proposed an automated runup-edge-tracking method for these recordings, demonstrating that runup time series and spectral characteristics similar to offshore measurements could be obtained, suggesting CCTV’s potential for tsunami detection. However, their method required somewhat complex, site-specific parameter tuning, which limited its broader applicability.

This study has two objectives. First, we present an almost automated runup-edge-tracking algorithm, designed for broad applicability by minimizing dependence on user-defined parameters. Video‐based runup monitoring approaches have existed for decades, yet many still require manual intervention^26^ or careful parameter tuning ^22^. They also often rely on analysis of a single cross-shore direction transect using time-stack images (e.g.,^27,28^), which ignores the issue of transect representativeness and is vulnerable to local noise. Our algorithm leverages multiple transects simultaneously, enabling a more robust runup time-series extraction compared to single-transect approaches, under diverse site and lighting conditions. Second, we apply the proposed method to the 2024 Noto tsunami recordings. By comparing CCTV-derived runup waveforms with numerical tsunami simulations and offshore observations, we assess the approach’s feasibility for this event.

## Methods

### Wave runup tracking method for CCTV footage

To develop and validate our approach under a wide range of weather and wave conditions—conditions not captured in the one-hour 2024 Noto event footage—we utilized a week-long CCTV dataset from the Hazaki Oceanographical Research Station (HORS) in Kamisu City, Ibaraki Prefecture. A camera mounted at approximately 9 m above mean sea level captured continuous 1280 × 720 px H.264 video at 25 fps (frames per second) between December 19 and 26, 2024, from which we extracted the selected representative segments, providing the extended dataset needed to assess robustness.

**Fig. 1:**
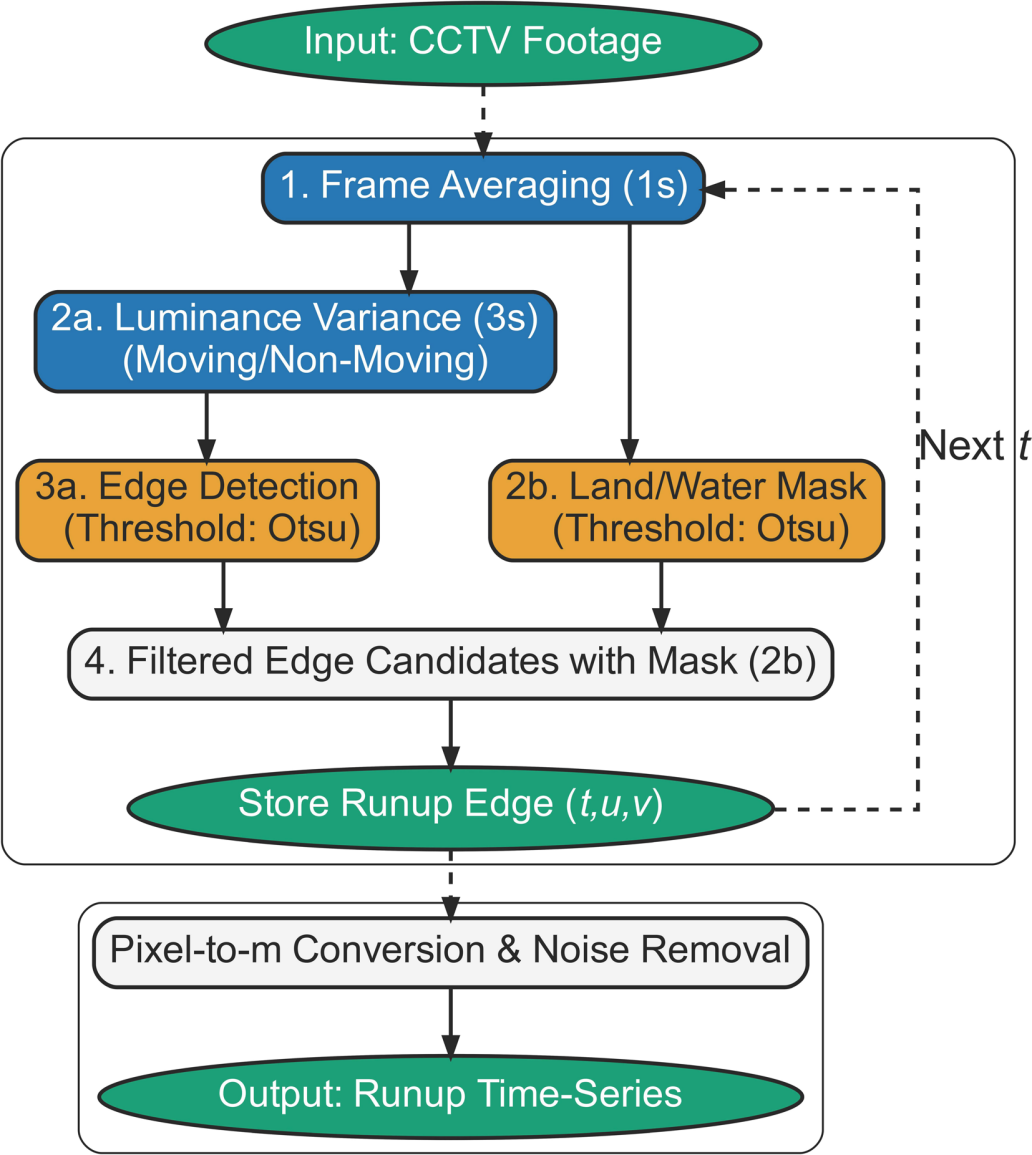
Flowchart of the proposed automated runup-edge tracking algorithm.

**Fig. 2:**
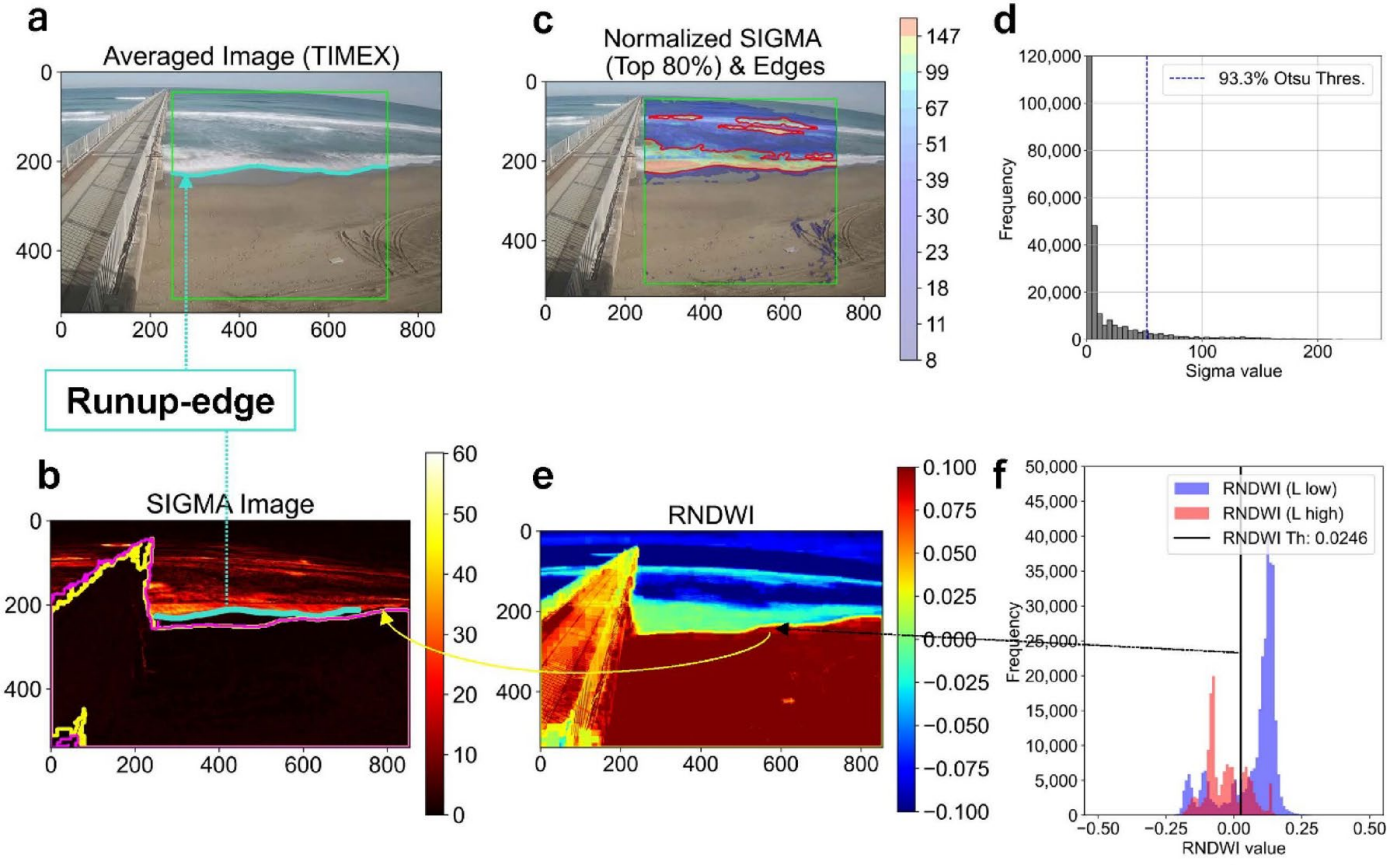
Overview of the proposed method. (**a**) Time-averaged (1 s window) image of the scene. Within the region of interest (ROI; green box), the shoreline candidate is marked by a turquoise dot—this pixel will become the “runup-edge.” (**b**) SIGMA image showing pixel-wise luminance standard deviation over consecutive TIMEX frames (3 s window). Wet–dry boundaries derived from the color mask (RNDWI; yellow) and CIEL *a*b*b* (magenta) are overlaid. (**c**) Normalized SIGMA image: the top 80% of normalized SIGMA values (color bar) are highlighted, and Canny-detected edges are drawn in red. (**d**) Histogram of normalized SIGMA values within the ROI. The blue vertical line marks the Otsu’s-based threshold used for image binarization in (c). (**e**) RNDWI image (RGB-based normalized difference water index) and (**f**) Histogram of RNDWI values inside the ROI. The black vertical line indicates the Otsu-derived wet–dry boundary (= 0.0246 in this case) used to mask out inland noise.

Our methodology (summarized by the flowchart in Fig. 1) first adapts two standard image processing techniques from coastal engineering: the Time-Exposure (TIMEX) image ^3,29^, which is an average of video frames, and the SIGMA image^30,31^, which represents the temporal variation (standard deviation in this study) of pixel luminance. Traditionally, both are computed over long periods (e.g., 10–15 min) to analyze wave-averaged conditions, such as the shoreline position or the extent of the surf zone. In contrast, our study applies these concepts over much shorter, consecutive time windows—${T}_{avg}$ for TIMEX and ${T}_{\sigma }$ for SIGMA (where ${T}_{avg}<{T}_{\sigma }$)—to resolve the dynamics of individual runup events. As illustrated in Fig. 2, this short-term TIMEX image effectively smooths small-scale dynamic noise, thereby making the runup edge more distinct. The corresponding SIGMA image visualizes uprush and backwash fluctuations as an “accumulated motion” image, clearly delineating individual runups (Fig. 2b).

On each normalized SIGMA image, we apply Gaussian smoothing to remove residual spatial noise. Within a user-defined region of interest (ROI), we binarize the smoothed SIGMA image using a threshold automatically determined by applying Otsu’s method^32^ to its histogram. We then run the Canny edge detector^33^ solely to extract the boundary of this binarized image as an edge (Fig. 2c, 2d). In our analysis, the resulting threshold typically fell within the 80th to 99th percentile range, effectively separating high- and low-SIGMA regions and thereby allowing us to track the runup edge at their boundary. Morphological closing and removal of small unconnected components then retain the principal runup-edge contour.

To extract a runup edge per frame, we scan each image row (or column) from landward to seaward and mark the first retained edge pixel as a candidate. Finally, a color mask (see Dynamic noise removal via color mask Section) removes remaining false positives (Fig. 2e, f) due to dynamic noise from moving objects such as pedestrians, yielding the definitive runup‐edge position for each frame.

Notably, most of the algorithm’s parameters exhibit minimal site dependence, and the only one that the user must explicitly set is the ROI (aside from the analysis transects). Moreover, provided that the aforementioned assumptions hold, the algorithm reliably tracked the runup edge at Hasaki under a wide range of brightness and wave conditions verified in our study (e.g., Supplementary Fig. S1, S2). Significantly, the proposed method could be applied directly to the Noto tsunami case without any site-specific tuning or modifications.

### Determination of TIMEX/SIGMA time windows

**Fig. 3:**
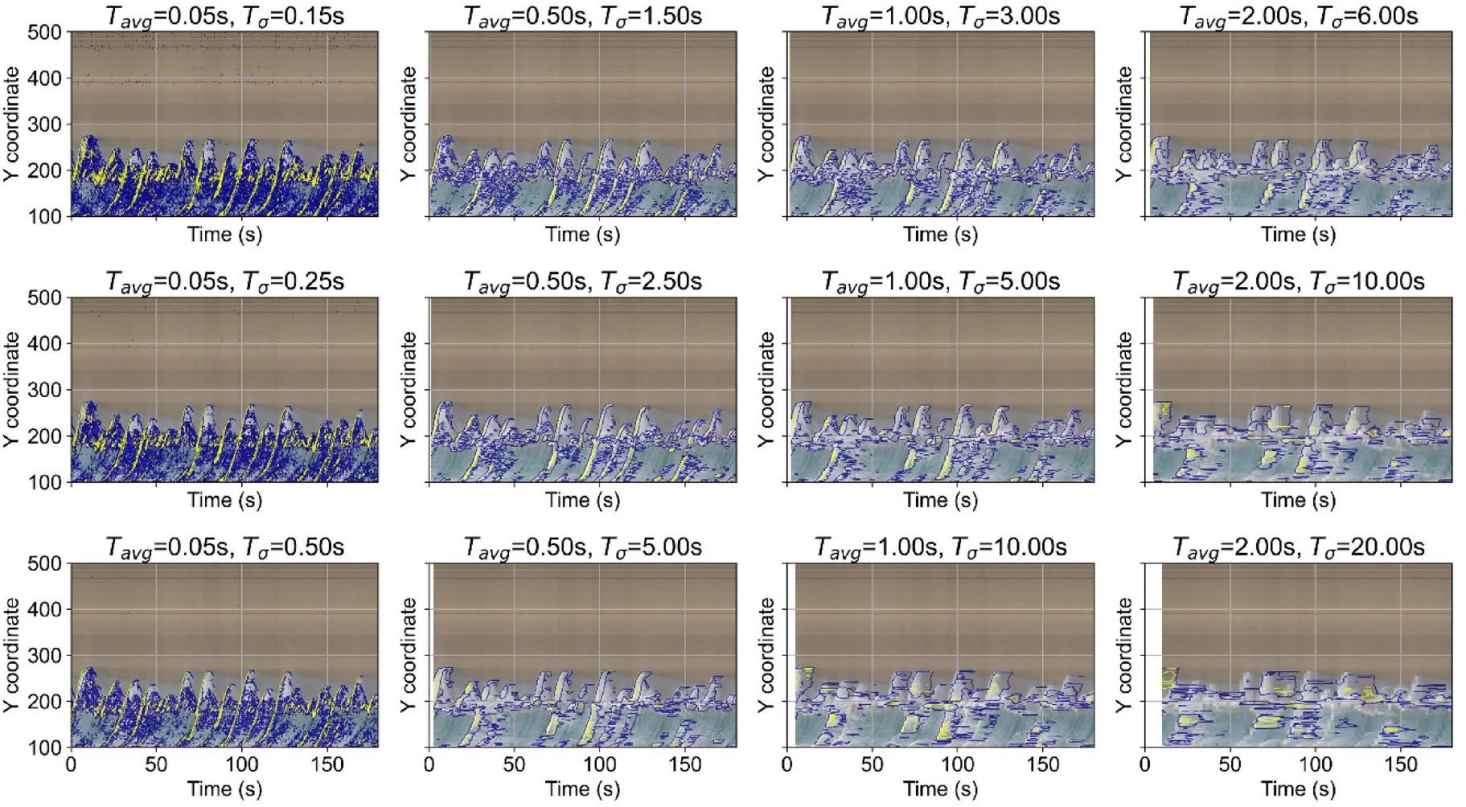
Time-stack images showing luminance variation along a transect at Hasaki Coast over a three-minute interval beginning at 12:00:00 on December 20, 2024. The blue and yellow curves trace, respectively, the 80th- and 99th-percentile luminance-variation levels on the transect at each time step. ${T}_{avg}$ and ${T}_{\sigma }$ represent the time windows used to compute the TIMEX and SIGMA images.

To resolve individual wave runup events, we used much shorter windows than the conventional 1–10 min^30,31^. As shown in Fig. 3, combining ${T}_{\sigma }$ of up to approximately one-half of the significant wave period (7.6 s) and ${T}_{avg}$ of one-third of ${T}_{\sigma }$ (with SIGMA computed over at least three consecutive TIMEX frames for robustness to noise) captured both uprush and backwash while smoothing land-side noise. When ${T}_{\sigma }$ was too long, swash details were averaged out (e.g., 10 s). Conversely, shortening the windows excessively increased noise, impaired shoreline detection, and multiplied the image count, thus prolonging processing time. Balancing these trade-offs, we applied ${T}_{\sigma }$= 3 s and ${T}_{avg}$=1 s for the 2024 Noto tsunami case, noting that the offshore significant wave period was ≈ 10 s.

### Dynamic noise removal via color mask

To eliminate dynamic noise on land (e.g., pedestrians, debris) detected in the SIGMA-based edge candidates, we filter those pixels using a color mask based on the R/B ratio (where R is pixel intensity in the red band, B is pixel intensity in the blue band in the RGB color space) or the b* channel of CIE L*a*b* space^34^. Over water, red light is strongly absorbed, yielding a low R/B ratio, whereas over sand or land the ratio is high, enabling clear separation of wet and dry regions^35^. Rather than raw R/B, we define an “RGB-based NDWI (Normalized Difference Water Index)” variant,

1 $$RNDWI = \frac{R - B}{{R + B}} = \frac{{\frac{R}{B} - 1}}{{\frac{R}{B} + 1}}$$

inspired by the MNDWI^36^ that is applied in the automatic estimation of the satellite derived shoreline^37^, and apply Otsu’s method^32^ to its bimodal histogram to determine an automatic threshold. The CIE L*a*b* b* channel provides luminance-independent chromatic contrast between water (blue) and land (yellow)^38,39^. In typical beach imagery, RNDWI and b* histograms behave similarly, but with a key difference stemming from their relationship to luminance: the CIE L*a*b* b* channel is largely independent of scene brightness, whereas RNDWI can be more sensitive to color changes in darker conditions (Supplementary Fig. S3). These masks remove most false‐positive candidates on the land side. Note that such visible‐light masks tend to identify the wet‐dry boundary revealed after maximum uprush, rather than the instantaneous runup front.

To ensure a high-quality analysis, the histograms for both the SIGMA image and the color mask are generated exclusively within the user-defined ROI. Since the color masks require a bimodal histogram, the ROI must include both land and water pixels without extreme imbalance; however, CCTV’s fixed viewpoint allows a one‐time ROI definition. Aside from this requirement, thresholds can be set automatically based on color information, enabling robust removal of dynamic noise in the relatively diverse footage from this study. Further robustness evaluations for pedestrians is also provided in the Supplementary Fig. S4.

### Application: the 2024 Noto Peninsula earthquake tsunami

**Fig. 4:**
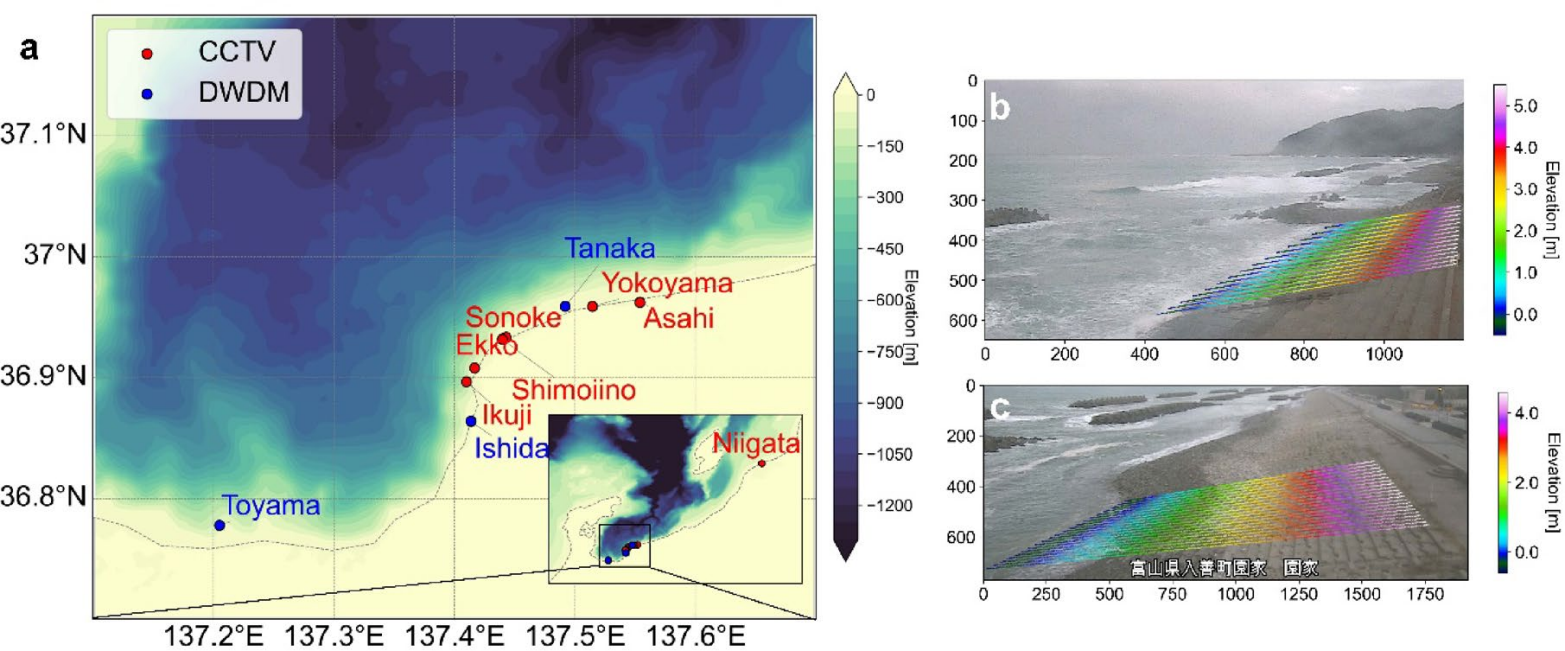
(**a**) Map showing the target area for the Noto event along with the CCTV (red dots) and DWDM (blue dots) (offshore observation) sites. Panels (b) and (c) show representative fields of view of the CCTV images (cropped for processing) at (**b**) Asahi and (**c**) Sonoke, as well as the transects used to generate the ensemble runup time-series. The pixels along each transect are assigned elevation values based on field surveys, and the shoreline positions are converted to runup heights (see Shirai et al.^22^ for details).

The 2024 Noto Peninsula tsunami was generated by an Mw 7.5 earthquake on 1 January 2024 at 16:10 JST, producing local inundation up to ~ 6 m near the epicenter^40^. We analyzed seven coastal CCTV footage from Toyama and Niigata prefectures (Fig. 4 and Supplementary Fig. S5), supplied by the Hokuriku Regional Development Bureau of the Ministry of Land, Infrastructure, Transport and Tourism (MLIT). All cameras recorded H.264-compressed Transport Stream (.ts) or MP4 video at 1920 × 1080 px and ~ 30 fps (one Niigata site at ~ 60 fps), enabling frame extraction via OpenCV in Python^41^. To validate our extracted runup time series, we incorporated 2 Hz wave observations from Doppler-type Wave Directional Meters (DWDM) at Toyama, Tanaka and Ishida. The analysis window was fixed from 16:00 to 17:00 JST, encompassing ~ 10 min before and ~ 50 min after the earthquake.

At the analyzed CCTV sites, a detached breakwater is present, and the two-dimensional nature of the wave field affected by the breakwater, as well as potential errors in runup edge tracking due to noise, may introduce errors in the tsunami period components of the runup waveform. Thus, by ensemble averaging the runup time series obtained from multiple transects, a final waveform is derived. The transect configuration is performed under the assumption that the slope along the seawall is flat (following ^22^), and no projective transformation is applied (Fig. 4). Although the number and spacing of transects is somewhat arbitrary, adjustments can be made by referring to the convergence behavior of error curves (e.g., runup height) as more transects are added. The accuracy evaluation, including the effect of the transect ensemble, is discussed in the Discussion section.

### Spectral and wavelet analyses

To characterize tsunami signatures at the CCTV sites, we first estimated the power spectral density (PSD) of the runup time series using Welch’s method (scipy.signal.welch^42^). Welch’s method is commonly used in tsunami research^43^. We applied a 1,024-sample Hann window with 50% overlap and zero-padding to 2,048 samples. These settings provide sufficient frequency resolution to distinguish the dominant tsunami and wind-wave bands. We then perform a continuous Morlet wavelet transform in MATLAB^44^, with scales from twice the sampling interval (2 s) upward and logarithmic increments *δj* = 0.025, computing 95% significance levels against red-noise backgrounds^45^. Wavelet analysis aids in separating and identifying tsunami components within the non-stationary runup time series.

## Results

### Time-series of runups and time-averaged power spectrum

**Fig. 5:**
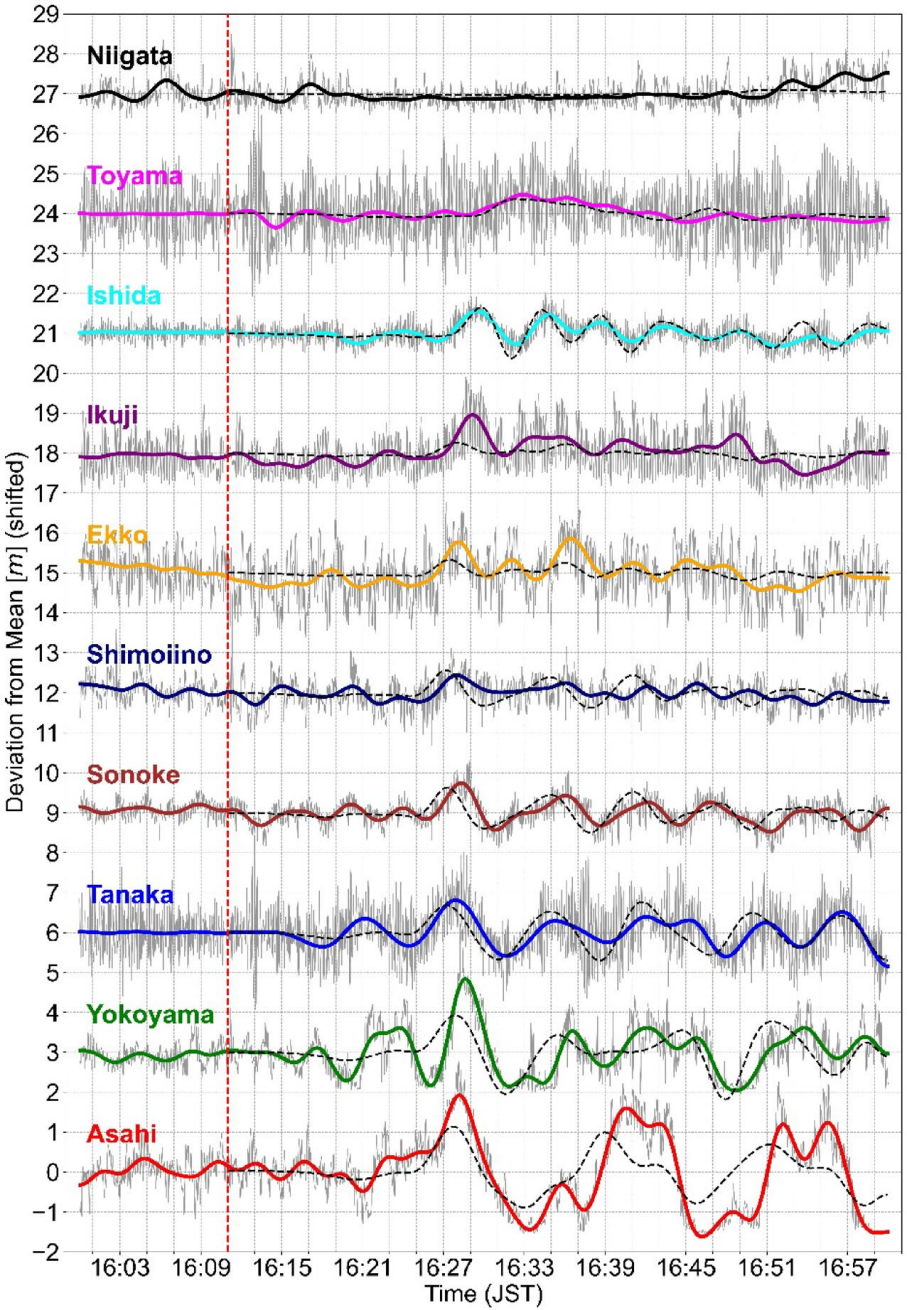
Time series from CCTV and DWDM sites between 16:00 and 17:00. For visual comparison of the waveforms, the vertical axis is offset by 3 m for each site, starting from 0 m. Each time series shows the deviation from its mean value during the analysis period. At each location, the waveform filtered with a fourth-order Butterworth low-pass filter (cutoff = 180 s) is shown in color, and the 5 s lowpass filtered original signal is plotted in gray. The black dashed line gives the numerical simulation output, initialized at the approximate tsunami onset (16:11:00, indicated by the red vertical line).

**Fig. 6:**
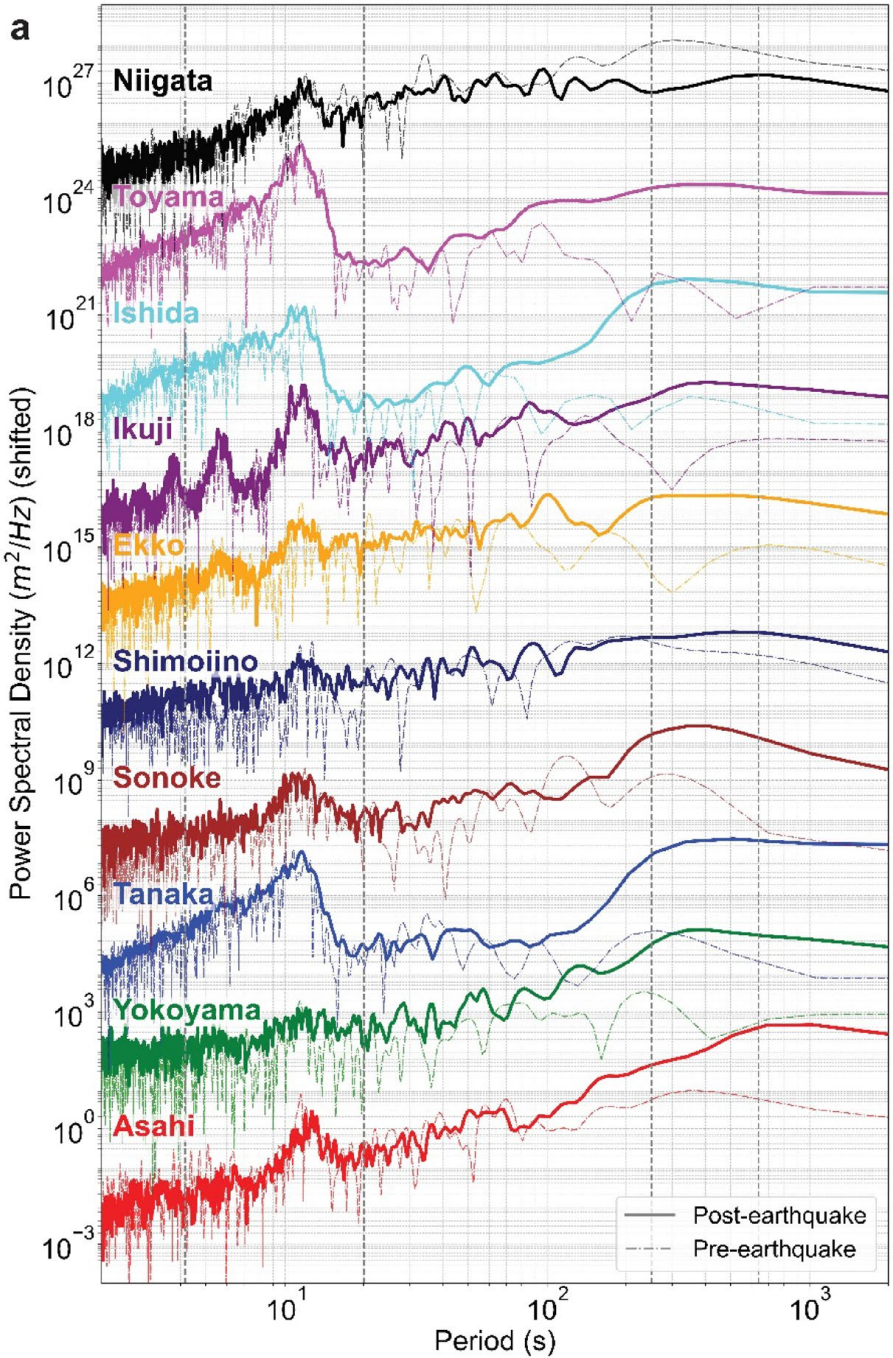

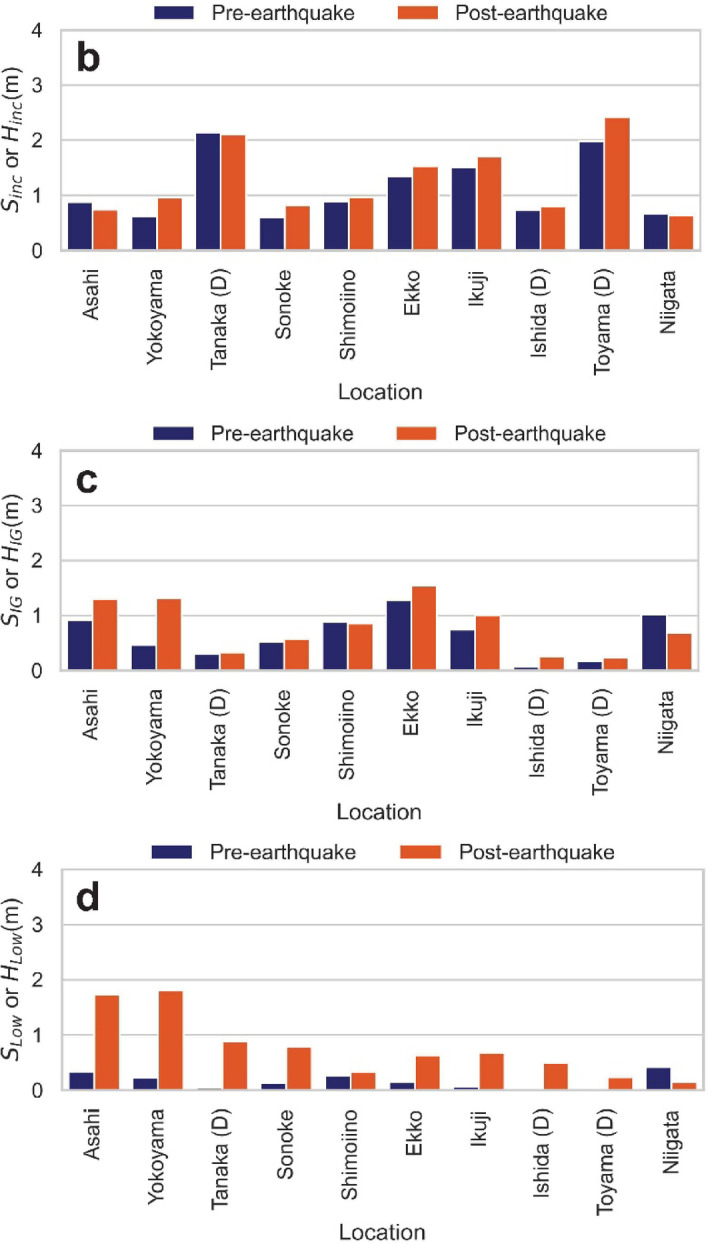
(**a**) Estimated power spectral density (PSD). The dash-dot line shows PSD estimated from the pre-earthquake data, and the solid line represents the post-earthquake data. For visualization purposes, the PSD values have been shifted vertically by a factor of 10^3^. (**b**–**d**) Spectral runup height or spectral wave height measured at each CCTV or DWDM location before and after the earthquake, in (b) the incident band, (c) the infra-gravity band, and (d) the low frequency band. Locations marked with (D) denote offshore DWDM sites.

Figure 5 presents the time series, and Fig. 6a shows the corresponding time‐averaged power spectra before and after the Mw 7.5 earthquake. In the runup series (Fig. 5), the first prominent positive peak appears around 16:28 JST at most sites in Toyama Prefecture, including both the CCTV and offshore DWDM locations—closely matching the leading‐wave arrival predicted by our numerical simulation (black dotted line in Fig. 5; details for numerical simulation is provided in Supplementary Text S1 and Table S1). It has been pointed out that under this event, prominent wind waves influenced the water levels observed in the coastal area^20^. A similar trend was observed in the runup time-series estimated in this study. At all sites, the maximum runup was caused by the runup of an individual wind wave occurring during the elevated coastal water level from the tsunami (visual confirmation is provided in the time-stack analysis in Supplementary Fig. S6). To quantify the tsunami energy content, we compute the spectral runup $S$ (or the spectral wave height $H$ for the offshore DWDM)

2 $$S = 4\sqrt {\mathop \smallint \limits_{a}^{b} E\left( f \right)df}$$

where $E\left(f\right)$ is the power spectral density, $f$ is the frequency, and the integration bounds (where *a* is the lower bound, *b* is the upper bound) is based on Senechal et al. (2011)^18^: the incident band ${S}_{inc}$ or ${H}_{inc}$ (0.05–0.24 Hz), the infragravity band ${S}_{IG}$ or ${H}_{IG}$ (0.004–0.05 Hz), and the low-frequency band ${S}_{Low}$ or ${H}_{Low}$ (0.0015625–0.004 Hz).

Figure 6b–d compares the changes in spectral runup/wave heights for each frequency band before and after the earthquake at each site. Caution is required when interpreting the changes in the incident and IG bands (Fig. 6b, c). For the former (incident band), the changes can be attributed to two main reasons: (i) phenomenological and (ii) systematic characteristics of the image processing. Reason (i) includes cases where the swash motion itself is altered by the tsunami, such as when changes in the effective foreshore slope due to tsunami-induced water level variations also alter the runup height^46^, or when incident waves mostly break at the detached breakwater at low water levels but not at high water levels (the latter was qualitatively observed in the footage at Asahi). For reason (ii), at sites where the swash is observed only on sloping seawalls (Asahi, Yokoyama, Ikuji), the seawater infiltrates very little after runup. This makes the landward edge of the backwash clearer compared to sandy or gravel beaches. Consequently, the instantaneous shoreline position tends to be captured as the maximum extent of the water rather than a typical swash motion, which can result in smaller amplitudes than the actual swash (especially at Asahi, see Supplementary Fig. S6).

On the other hand, for the latter (IG band), the changes before and after the earthquake are considered to be predominantly caused by phenomenological factors. This is because the systematic characteristics of the method, as described in (ii), primarily affect the incident band components. However, at some sites (the latter half of the time-series at Yokoyama, and Niigata), there were slightly more misdetections during image processing compared to other sites, which sometimes distorted the IG band components. These shoreline misdetections mostly occurred not on the landward side where the color mask was applied, but rather on the seaward side where brightness fluctuations temporarily became large, such as at breaking wave locations.

Finally, the changes in ${S}_{Low}$ before and after the earthquake are considered to be least affected by high-frequency noise originating from image processing compared to the other bands. Therefore, the significant amplification of power in this band serves as a robust and direct indicator of the tsunami’s arrival and its sustained presence at the coast.

Table 1 summarizes the post‐ to pre‐earthquake amplification factors of the low-frequency band (${S}_{Low}$) for each site. Offshore DWDM observations show that ${S}_{Low}$ was amplified by factors of 37.4 at Ishida, 19.9 at Tanaka, and 12.9 at Toyama. A similar multi-fold (> 1) increase was observed in the CCTV-derived runup series at most locations. This indicates that, for this tsunami event, our analysis method successfully detected the energy amplification associated with the tsunami’s arrival from the shoreline observations. The Niigata site, on the other hand, presents a distinct case; the tsunami arrived later and with a smaller amplitude compared to the sites in Toyama Prefecture. Nevertheless, the CCTV-derived runup waveform successfully captured the tsunami-induced water level rise predicted by numerical models between 16:40 and 16:50 JST. However, the post- to pre-earthquake ratio of its ${S}_{Low}$ component was less than 1, making it an outlier compared to the other sites (Table 1). Therefore, to ensure the reliability of the analysis, the Niigata site was excluded from subsequent analyses. It is also worth noting that the ${S}_{IG}$ exhibited a significant mean amplification of 37% (Fig. 6c) , while the amplification of the ${S}_{inc}$ was less pronounced (15% on average). The next section uses wavelet analysis to examine the temporal evolution of these frequency components in more detail.

**Table 1:** Post- to pre-earthquake amplification factors of the low-frequency component (${S}_{Low}$)

Location	Asahi	Yokoyama	Tanaka (D)	Sonoke	Shimoiino
Amplification Factor	5.1	7.8	19.9	6.1	1.3
Location	Ekko	Ikuji	Ishida (D)	Toyama (D)	Niigata
Amplification Factor	4.2	9.9	37.4	12.9	0.4

Sites marked with (D) denote offshore DWDM observations; all others are derived from coastal CCTV

### Wavelet analysis result

**Fig. 7:**
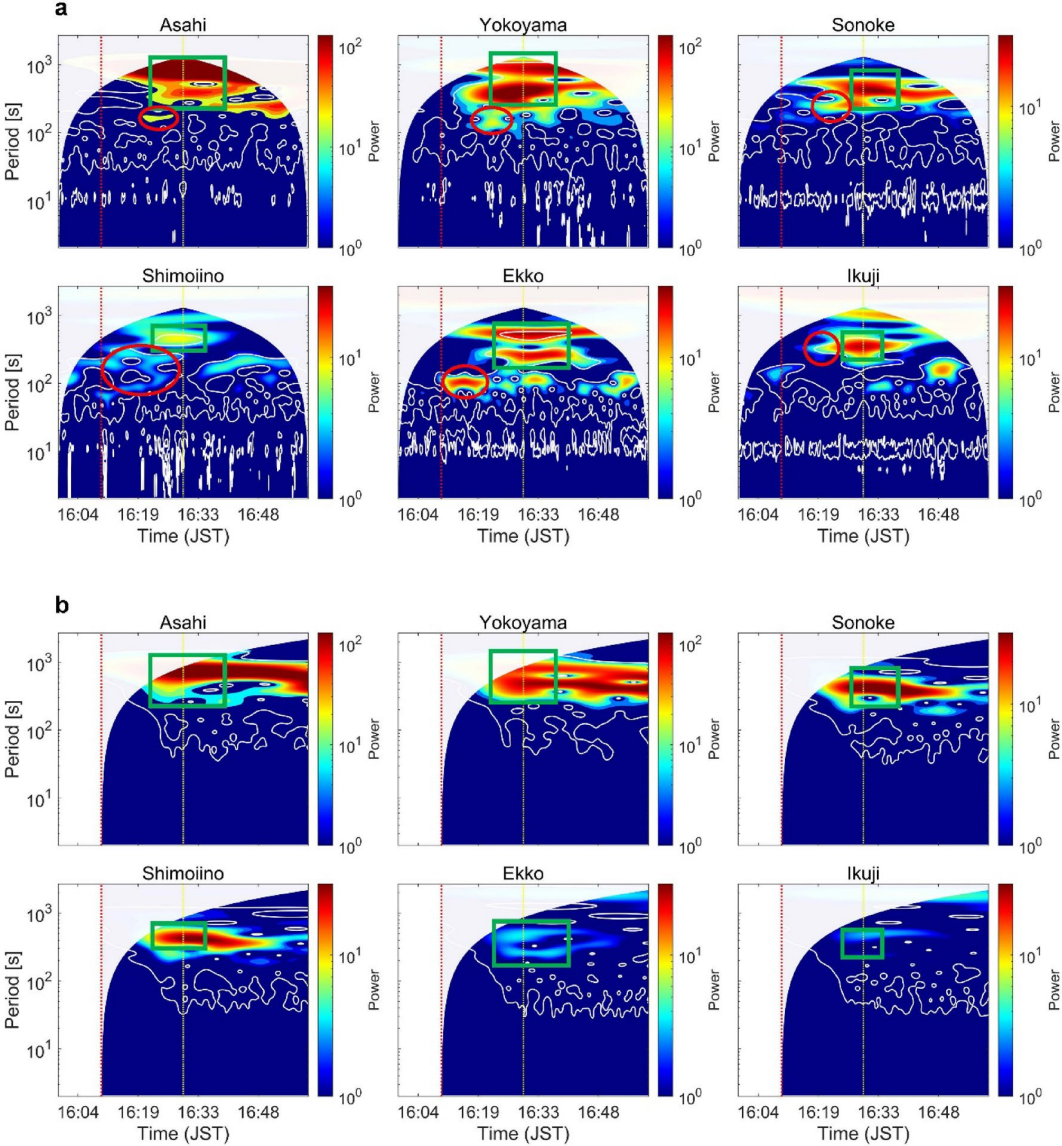
(**a**) Continuous wavelet power spectra of CCTV-derived runup height time series. Top row (left to right): Asahi, Yokoyama, Sonoke; bottom row (left to right): Shimoiino, Ekko, Ikuji. The red and yellow vertical dotted lines mark the earthquake onset (16:10:23) and 16:30:00, respectively. The white-shaded area denotes the cone of influence (COI), and white contour lines indicate the 95% significance level. Within the green rectangle, the arrival of the first leading wave from the fault tsunami is consistently captured across all sites; the red circle highlights long-period oscillations (~ 100–300 s) whose origins may not be fully explained by the fault tsunami alone. (**b**) Same as (a), but wavelet spectrogram of the numerical simulation results.

Figure 7a shows the wavelet spectrogram of the extracted runup series at the six sites. The first significant arrival of tsunami energy is observed roughly 20 min after the earthquake, a timing consistent with the initial observation in the runup time-series (Fig. 5). A coherent energy band at 300–500 s is visible at all sites, aligning with the dominant periods identified in the PSD analysis (Fig. 6a). All sites also exhibit a distinct energy component at periods longer than 500 s. This spatial uniformity confirms that the initial fault‐induced tsunami carried periods in the 300–500 s range, with additional longer‐period energy.

To verify the origin of these signals, we applied the same wavelet analysis to the numerical simulation (Fig. 7b). The simulation reproduces the 300–500 s band at all sites, as well as the > 500 s component at most sites, confirming their fault–rupture tsunami source. However, the longer‐period (> 500 s) components lie near the edge of the one‐hour record and intrude into the cone of influence (COI), so they may be affected by edge artifacts and should be interpreted with caution (especially in the runup series).

The simulation also captures the post-quake increase in the IG band, indicating that the observed IG-band amplification in the runup series cannot be attributed solely to wind‐wave runup but includes fault–rupture tsunami energy.

At Ekko, a distinct 100–200 s period component emerges around 700 s post-quake—absent from the fault-only simulation—suggesting a possible submarine-landslide tsunami source^20,21^. At Shimoiino, however, the 100–300 s band is already present before the earthquake, precluding unambiguous attribution to the tsunami. At the remaining sites (Asahi, Yokoyama, Sonoke, and Ikuji), the post-quake appearance of this band could likewise reflect submarine-landslide activity, but its spectral overlap with fault-induced components prevents definitive separation with the current dataset.

## Discussion

### Accuracy of the runup height time-series and efficacy of transect ensemble

In this study, we developed and applied a new runup‐tracking method to 2024 Noto CCTV footage. The extracted runup height time-series reproduce offshore tsunami spectrum, demonstrating that the method provides a reliable basis for tsunami detection in the cases studied. To further validate these results, we compared the CCTV-derived series against manually traced runup waveforms and performed a detailed accuracy evaluation. Expert-traced shoreline positions at 10 s intervals served as ground truth; these positions were digitized directly on the original images and then averaged over all transects. We also examined the stability of the ensemble approach, considering the risk that any single transect might not be fully representative of the overall swash motion or could be contaminated by localized noise, which would degrade the accuracy of a single-transect measurement.

Table 2 summarizes the accuracy evaluation for Yokoyama and Ekko. At Yokoyama, after removing wind‐wave components and short‐duration noise with a 30 s low-pass filter, the RMSE is 0.177 m (RMSE/σ = 0.335, where σ is the standard deviation of the manually traced runup heights) and the correlation coefficient is 0.949. This indicates that IG-band and lower-frequency runup fluctuations are extracted with high fidelity. Applying a 180 s low-pass filter, which removes large part of the IG energy, reduces the RMSE to 0.094 m (RMSE/σ = 0.200) and raises the correlation to 0.982, showing that the method achieves relatively high accuracy at this site in capturing low-frequency, mean water-level changes that represent the tsunami runup.

**Table 2:** Accuracy evaluation results of runup height time series waveforms extracted from CCTV (Yokoyama and Ekko)

	Yokoyama			Ekko		
Cutoff [s]	RMSE [m]	RMSE/$\sigma$	Correlation	RMSE [m]	RMSE/$\sigma$	Correlation
30	0.177	0.335	0.949	0.421	0.744	0.676
60	0.140	0.271	0.966	0.325	0.684	0.734
120	0.110	0.222	0.977	0.242	0.644	0.765
180	0.094	0.200	0.982	0.191	0.567	0.824
240	0.084	0.189	0.983	0.166	0.523	0.853
300	0.070	0.171	0.986	0.147	0.487	0.877

“Cutoff” indicates the cutoff period of the low-pass filter applied to the waveform. σ represents the standard deviation in runup heights over the entire time series of manually extracted waveforms, “RMSE” indicates the root mean squared error of runup height of the CCTV-extracted waveforms relative to the manually extracted ones, and “Correlation” denotes the correlation between the two sets

At Ekko, the larger runup distance within the image makes waveform extraction more error-prone (Supplementary Fig. S5). This is mainly because runup edge during backwash is less distinct than during uprush, introducing uncertainty into the ground truth itself. For the 30 s LPF waveform, the RMSE is 0.421 m (RMSE/σ = 0.744) with a correlation of 0.676. With a 180 s LPF, the RMSE falls to 0.191 m (RMSE/σ = 0.567) and the correlation rises to 0.824. Although extraction errors at Ekko are larger than at Yokoyama, the tsunami-period signal remains clearly observable.

Supplementary Figure S7 shows how RMSE and Pearson’s correlation for the 180 s LPF runup series change as more transects are added to the ensemble. At Yokoyama, sampling 1,000 random subsets from 31 transects reveals that RMSE falls steeply with the first 5–6 transects and then stabilizes by about 10, while correlation increases in tandem. At Ekko, where only 8 transects were defined, evaluating every combination produces a similar pattern: RMSE drops sharply with 2–3 transects and converges by about 5, reflecting lower variability among its lines. These analyses highlight the strength of the ensemble approach. While any single transect carries the risk of not representing the mean tsunami-induced water level fluctuation, our results show that a stable and representative measurement of this fluctuation was achieved in our cases by averaging a relatively small number of transects. Moreover, by averaging across an ensemble that includes multiple transects, the influence of localized noise and occasional outliers is diluted, thereby improving the reliability of the extracted runup waveform. In real-time detection—where it is difficult to know in advance which single transect may fail—this transect-ensemble approach could be a promising one.

### Identification of fault-induced tsunami components in the runup waveform

Spectral and wavelet analyses yield three principal findings. First, the fault-induced tsunami’s first leading wave—arriving at approximately 16:28 JST—produced a remarkable runup component with a period of 300–500 s (with some longer-period oscillations also evident), consistently observed at all CCTV sites in Toyama Prefecture. Second, numerical simulations confirm that these periods also appear in offshore tsunami waveforms. Third, after the earthquake the IG band amplitude in the CCTV-derived runup series increased by a factor of 1.37. According to our wavelet analysis in that band, the runup signal appears to include at least three components: one derived from the fault-induced tsunami (confirmed by numerical simulations), one attributable to wind waves already present before the quake, and one possibly linked to a submarine-landslide tsunami^20,21^.

At sites such as Asahi and Yokoyama, where the IG-band increase was particularly pronounced, the underlying causes remain unclear. One possible explanation is that tsunami-driven rises in mean water level altered the effective beach slope, as mentioned by Matsuba et al.^46^; however, confirming this hypothesis at our complex study sites, which feature coastal structures such as breakwater, was beyond the scope of the present work. More broadly, understanding how wind waves influence tsunami runup is critically important from an engineering standpoint and may help to refine our interpretation of the results; this therefore represents a key area for future research.

### Usefulness of CCTV-extracted runup time-series in tsunami detection

Building on our identification of tsunami-related components in the CCTV-derived runup series, we next consider its potential for practical tsunami detection.

Regarding processing time, which is critical for real-time applications, we benchmarked the proposed method (${T}_{avg}$ = 1 s and ${T}_{\sigma }$ = 3 s) on a modern desktop CPU (Intel Core i7-14700 with 64 GiB RAM) (Supplementary Fig. S8). Excluding the time required to write out captured video files and to transfer them to the analysis environment, execution time scaled with the post-trim frame size: at Yokoyama (frames trimmed to 16.7% of full frame), processing ran at 24% of real time; at Sonoke (71.3% trimmed), it took 87% of real time; other sites fell between these extremes. These performances could potentially be improved with code optimizations. While the processing performance is acceptable for real-time applications, several limitations of the proposed method must be carefully considered.

In terms of the image analysis, potential limitations of the proposed method include that, to date, it has been validated only at Hasaki and for the Noto event, so further verification across varied locations and tsunami types is needed. Caution is also warranted under unanticipated visual conditions, where extreme scenes dominated by moving vehicles, animals, or crowds can overwhelm the color mask and cause substantial misdetections. Both RNDWI and the Lab b* channel may mistake land objects of sea-like color for water, and shadows can cause misdetection of land–sea boundaries (Supplementary Fig. S9). In these cases, SIGMA-based edge detection often remains accurate, but faulty masking sharply increases the risk of false positives, indicating that some sites may require customized masking strategies to mitigate shadow and color artifacts. These issues reflect the inherent limitations of rule-based systems: they offer transparent behavior and ease of interpretation but may struggle under visually complex conditions. Incorporating machine learning or deep learning to distinguish runup from moving objects and to enhance noise detection and removal could further strengthen robustness and broaden applicability.

Additionally, we assumed that foreshore slopes along each transect were known in advance, but on highly dynamic coasts—such as sandy beaches with daily seabed changes—transect positions will require regular updating. To address this, our method could be combined with traditional camera-based bathymetric surveys^47,48^ or multi-sensor setups incorporating LiDAR^49^ to help maintain detection accuracy over time. Additionally, although not implemented here, automating adjustments for camera rotation and other view-angle changes will be important for robust, long-term operation.

Physically, a key limitation of this study is that the proposed method remains unvalidated for tsunami characteristics different from those observed at our study sites during the Noto event. This includes larger tsunamis that form bores and lead to inundation, as well as tsunamis that approach the shoreline at an oblique angle. For example, according to Pedersen^50^, the maximum runup height for an obliquely incident tsunami is expected to decrease as the angle of incidence increases, with a reduction of about 12% estimated for an incidence angle of 45° compared to normal incidence. Therefore, when estimating offshore tsunami wave height from the maximum runup, it is also important to consider the two-dimensional nature of the tsunami.

Finally, runup waveforms are strongly influenced by IG-band development and local bathymetry, resulting in complex signals that make it difficult to completely isolate tsunami-specific components in our case. Nevertheless, as suggested by our results, the runup height record can serve as a valuable indicator of tsunami arrival and energy. Refining signal separation methods to disentangle overlapping contributions from fault-induced waves, submarine-landslide tsunamis, and wind-wave-induced long period components should improve its reliability as a detection criterion.

Despite current technical constraints and remaining challenges, the proposed method delivers reliable runup-edge tracking, typically processes faster than real time, and requires minimal site-specific tuning, all of which are desirable characteristics for real-time applications with high versatility and ease of deployment. This study provides the first benchmark for CCTV-derived runup time-series tsunami detection, and with further validation, advanced signal separation, and machine-learning integration, can support its transition into real-world use.

## Conclusion

We have developed an almost fully automated method for monitoring wave runup with ordinary coastal CCTV cameras. The algorithm builds on the observation that, in typical beach scenes where runup occurs, the wave runup edge produces one of the strongest frame-to-frame luminance changes. This change appears as a bright feature in a short-term luminance-variation (SIGMA) image. By extracting that feature and applying a simple binary mask based on the different visible-light reflectance of land and water, the method tracks the runup edge while requiring only minimal user input. Extensive tests at Hasaki Coast confirmed stable performance under varied sunlight, wave height and transient obstacles.

Applied to the 1 January 2024 Mw 7.5 Noto Peninsula tsunami footage, the algorithm reproduced the tsunami’s dominant 300–500 s (and longer periods) energy band and reached site-specific accuracies of RMSE = 0.094 m at Yokoyama and 0.191 m at Ekko after low-pass filtering (cut-off = 180 s), while processing ran one to four times faster than real time on a standard desktop computer. These findings suggest that automatically extracted CCTV runup time-series can serve as a practical, low-cost complement to offshore gauges for both real-time tsunami detection and post-event reconstruction—particularly in areas where in-situ sensors are sparse.

Despite these promising results, this study has key limitations. The method has been validated on only one tsunami event—the relatively small Noto tsunami—and its performance on larger, bore-forming tsunamis that cause inundation remains unverified. These limitations define the critical next steps for future work. Future efforts will therefore focus on validating the method across a broader range of tsunami events and coastal topographies. Concurrently, we will continue to refine the signal-separation step to better distinguish between different long-wave sources, such as fault-generated tsunamis, submarine-landslide tsunamis, and wind-wave infragravity motions. Addressing these challenges is essential to further improve reliability and move this approach toward operational deployment.

## Supplementary Information

Below is the link to the electronic supplementary material.

Supplementary Material 1

## Data Availability

Hasaki CCTV footage is publicly available on YouTube: https://www.youtube.com/@HORS_Hasaki/streams. The sample analysis source code is available from the corresponding author on reasonable request. Due to rights restrictions, the 2024 Noto tsunami CCTV recordings cannot be made publicly available. NOWPHAS wave data are available from the MLIT archive at https://www.mlit.go.jp/kowan/nowphas/.
