# Supplementary material for: Automated wave runup monitoring using coastal CCTV cameras for tsunami detection
Source: Sci Rep. 2025 Nov 29;15:44429. doi: 10.1038/s41598-025-28874-x (PMC12738557; doi:10.1038/s41598-025-28874-x)
Supplement: Supplementary file 1 — Supplementary Material 1 [file 41598_2025_28874_MOESM1_ESM.docx]

Supplementary Information for

Automated Wave Runup Monitoring Using Coastal CCTV Cameras for Tsunami Detection

Tomoki Shirai^1^ and Taro Arikawa^2^

1 Research Fellow (DC2), Japan Society for the Promotion of Science (JSPS); Graduate School of Science and Engineering, Chuo University, 1-13-27 Kasuga, Bunkyo-ku, Tokyo, 112-8551, Japan.

2 Department of Civil and Environmental Engineering, Chuo University, 1-13-27 Kasuga, Bunkyo-ku, Tokyo, 112-8551, Japan.

Corresponding Author: Tomoki Shirai (a17.rc4x@g.chuo-u.ac.jp)

Contents

Figure and figure caption S1-S9

Text S1

Table S1


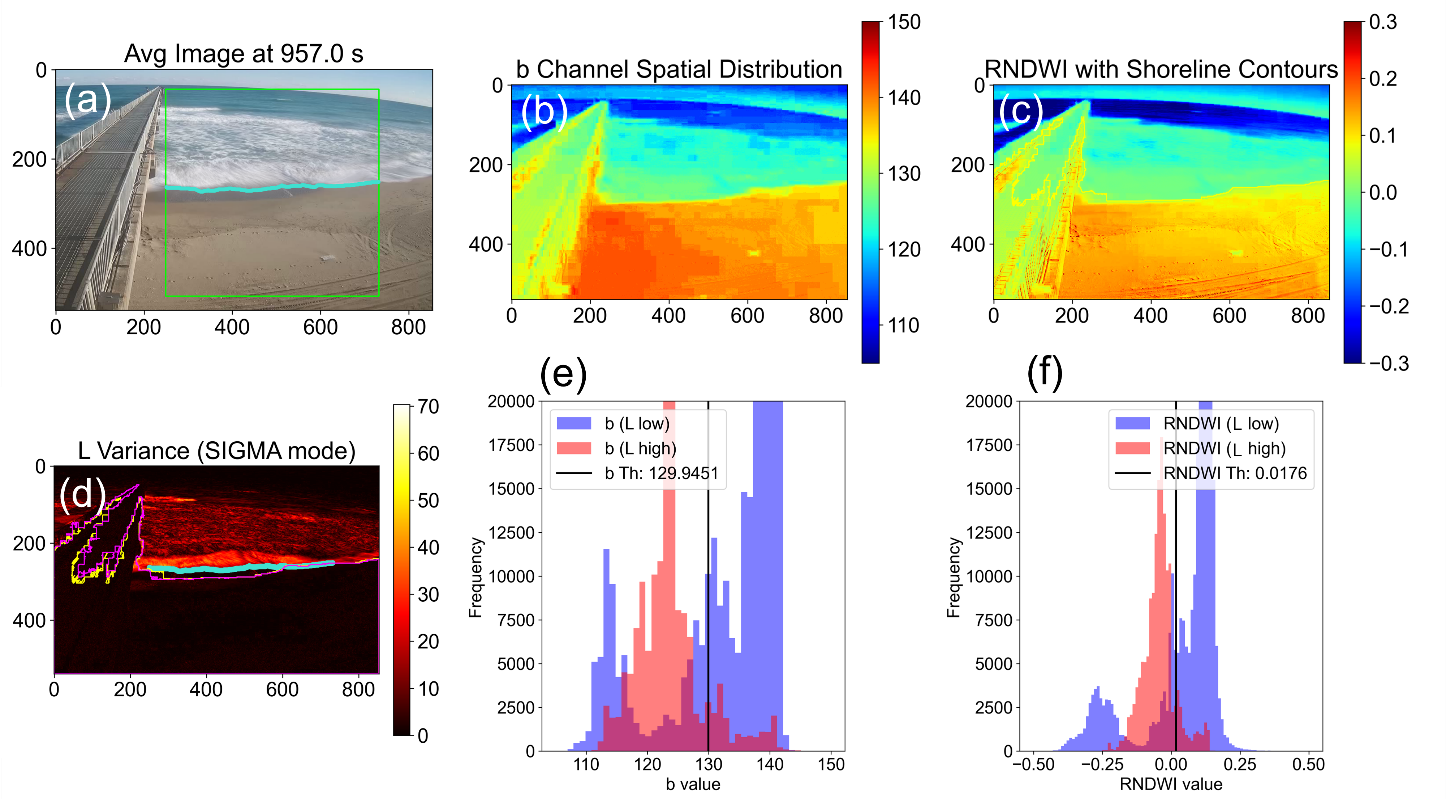


**Figure S1**. Example of shoreline (runup-edge) extraction under strong sunlight and high-wave conditions at HORS (∼09:00 on 22 December 2024). (a) TIMEX image (1 s window) with the extracted shoreline (turquoise) and the ROI (green box). (b) CIEL*a* b* b* channel. (c) RNDWI image. (d) SIGMA image (3 s window) overlaid with the shoreline (turquoise) and land/water boundaries from b* (magenta) and RNDWI (yellow). (e) b* histogram and Otsu threshold used to define the land-sea boundary (black vertical line). (f) Same as (e) but for RNDWI.


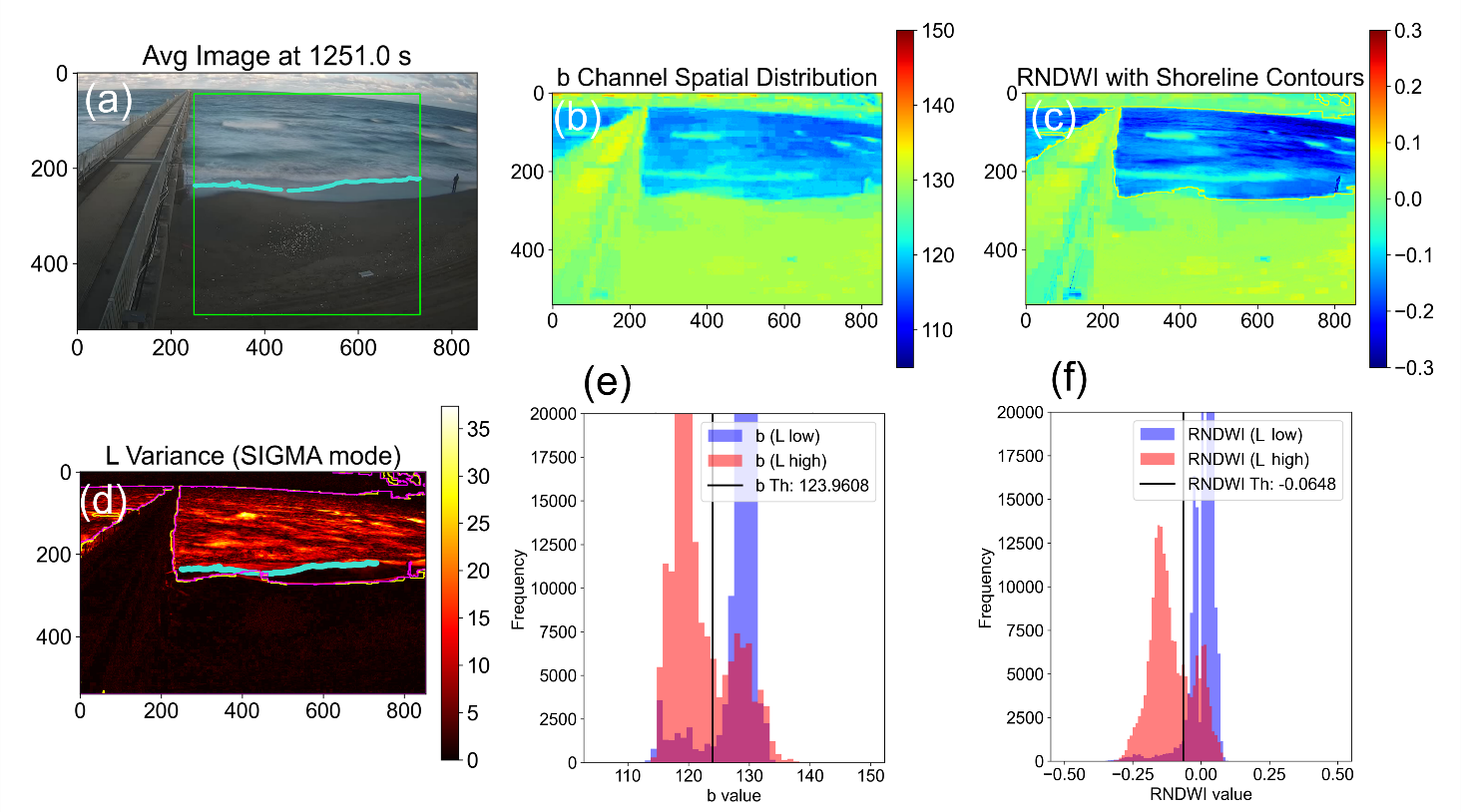


**Figure S2.** Same as Figure S1 but under low-light and calm-wave conditions (~15:00 on 18 December 2024).


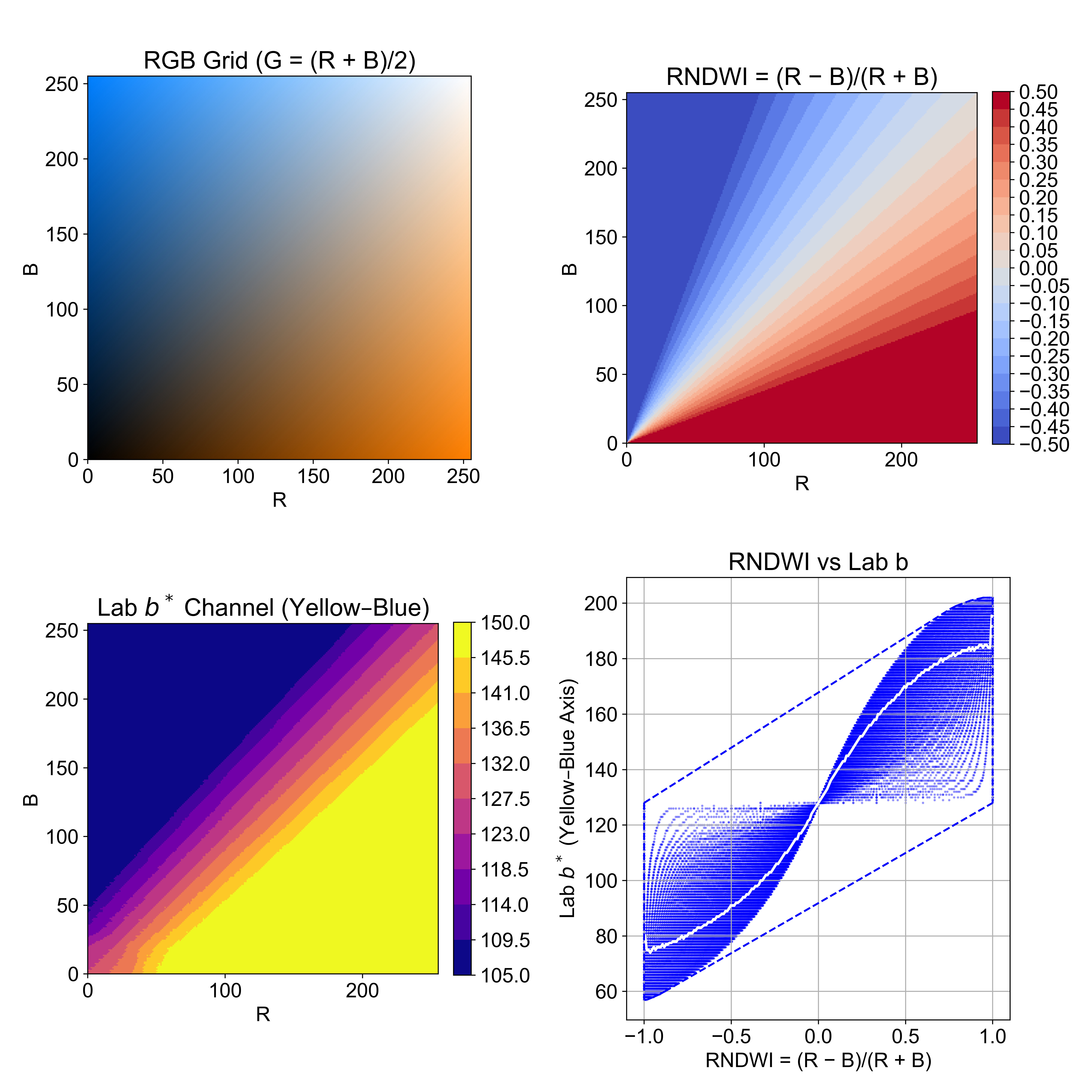


(b)

(a)

(d)

(c)

**Figure S3.** Sensitivity of the color mask luminance using an ideal beach image. (a) Input RGB image, where darker conditions are shown in the lower left and brighter conditions in the upper right. Ideally, the shoreline lies on the 45° line (where the order of red and blue is reversed). (b) RNDWI image and
(c) b* channel image. (d) Relationship between RNDWI and b* across the entire input image from (a). The white line denotes the median b* value for each RNDWI value. An ideal shoreline position corresponds to either b* = 128 or RNDWI = 0.


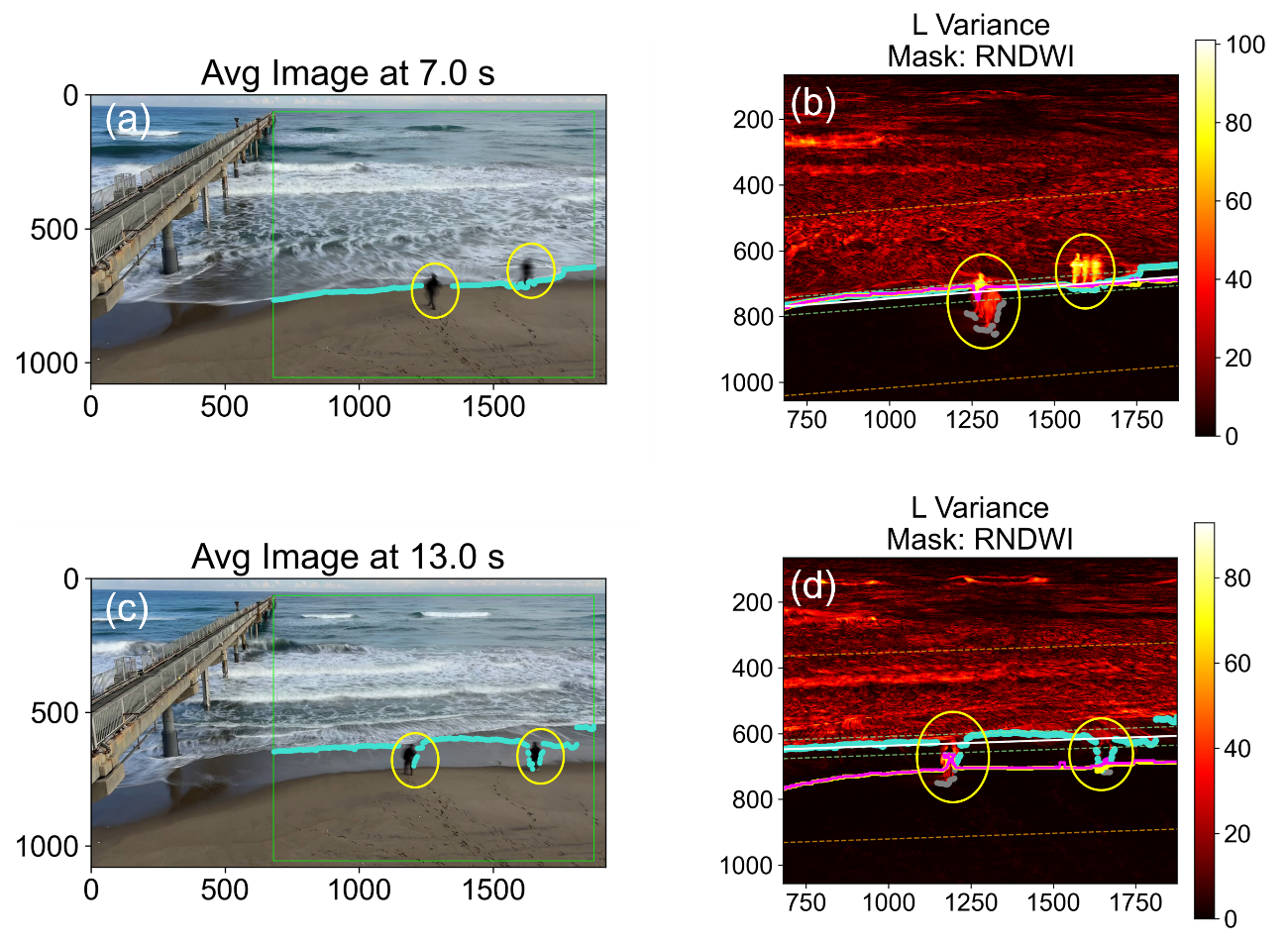


**Figure S4.** Example of color mask behavior when pedestrians are present as noise. (a) and (b) In these panels, the pedestrian on the left is detected as a dynamic region in the sigma image (b); however, since the pedestrian is located outside the land/water boundary, the one is excluded from the shoreline position in (a). (c) and (d) In this example, a gap appears between the runup edge, and the land/water boundary produced by the color mask, and a person is detected within that region. Nevertheless, on the land side, pedestrian noise is still effectively removed from the shoreline candidate positions.

**
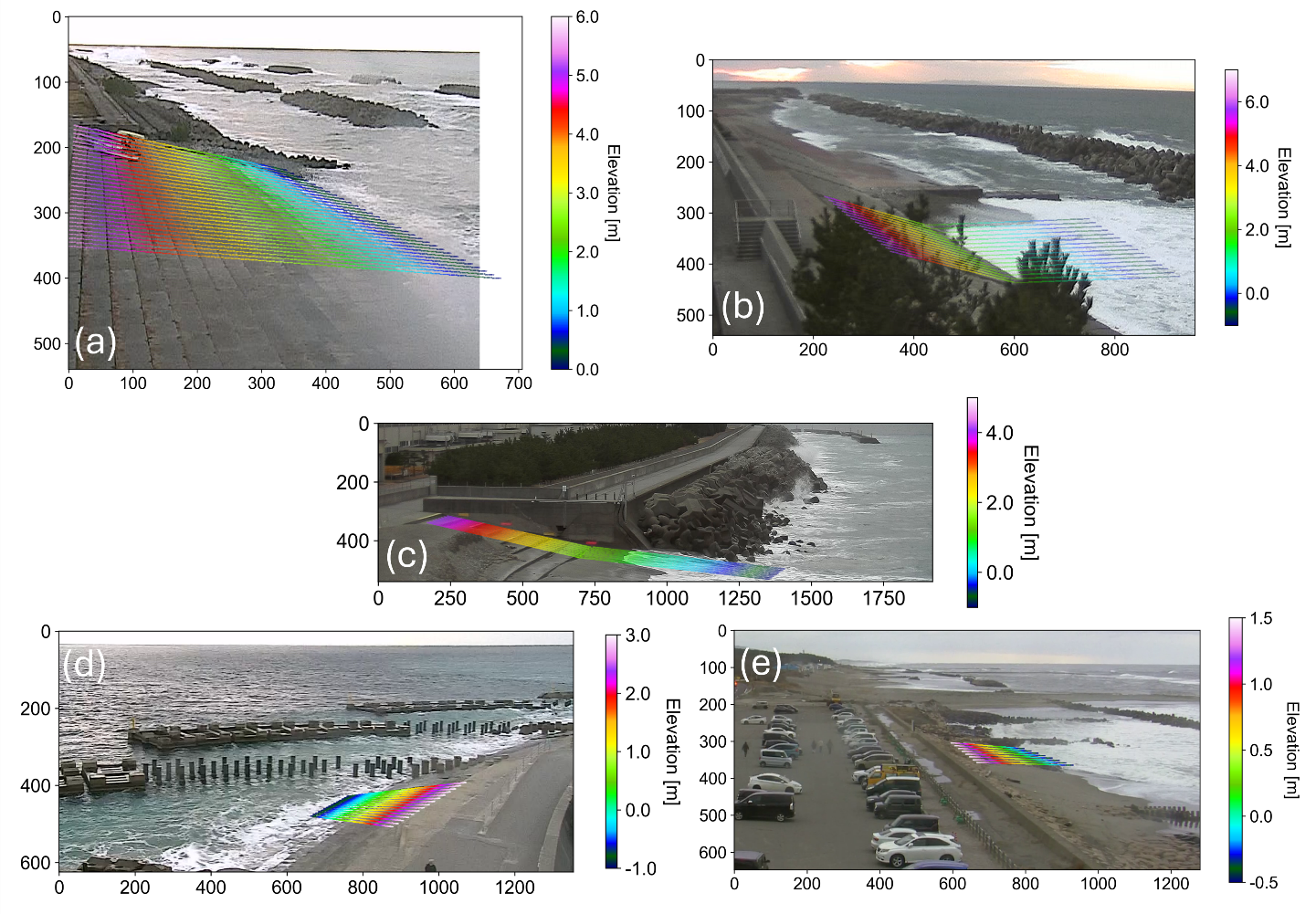
**

**Figure S5.** The CCTV field of view, defined transects, and pixel elevations (color scale) along each transect for the five locations not shown in Fig. 4: (a) Yokoyama, (b) Shimoiino, (c) Ekko, (d) Ikuji, and (e) Niigata. Note that the transect definitions for Yokoyama, Shimoiino, and Ekko are the same as in Shirai et al. (2024).

**
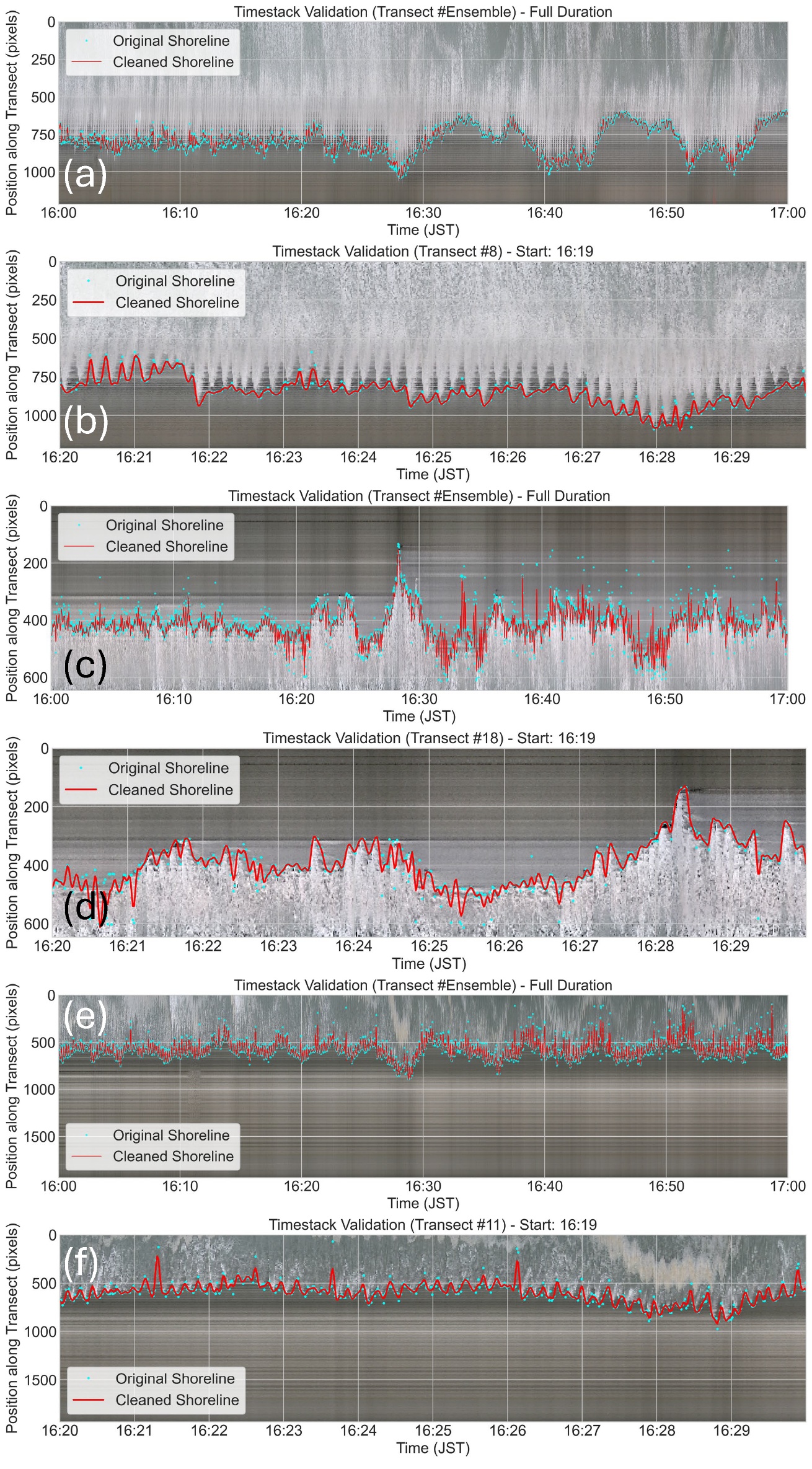
**

**Figure S6.** Time-stack images for each CCTV site with the extracted runup time series overlaid. Panels (a), (c), (e), (g), (i), (k), and (m) show the full one-hour duration (16:00–17:00 JST), where the transect-ensemble time series is overlaid on a time-stack image generated from a representative near-central transect for each respective site: Asahi, Yokoyama, Sonoke, Shimoiino, Ekko, Ikuji, and Niigata. The adjacent panels (b), (d), (f), (h), (j), (l), and (n) show focused 10-minute views of the single-transect time series from the same respective central transects. These focused views cover the 16:20–16:30 JST interval, which includes the first prominent peak (~16:28 JST), with the exception of Niigata (n), which shows 16:50–17:00 JST due to its later arrival time. In all panels, turquoise dots are the raw extracted runup positions, while the red line is the 5 s low-pass filtered time series, which removes high-frequency noise, primarily at periods shorter than the wind-wave band. Note that for Ikuji (k, l), the camera’s field of view was altered at approximately 16:11 and 16:47 JST.

**
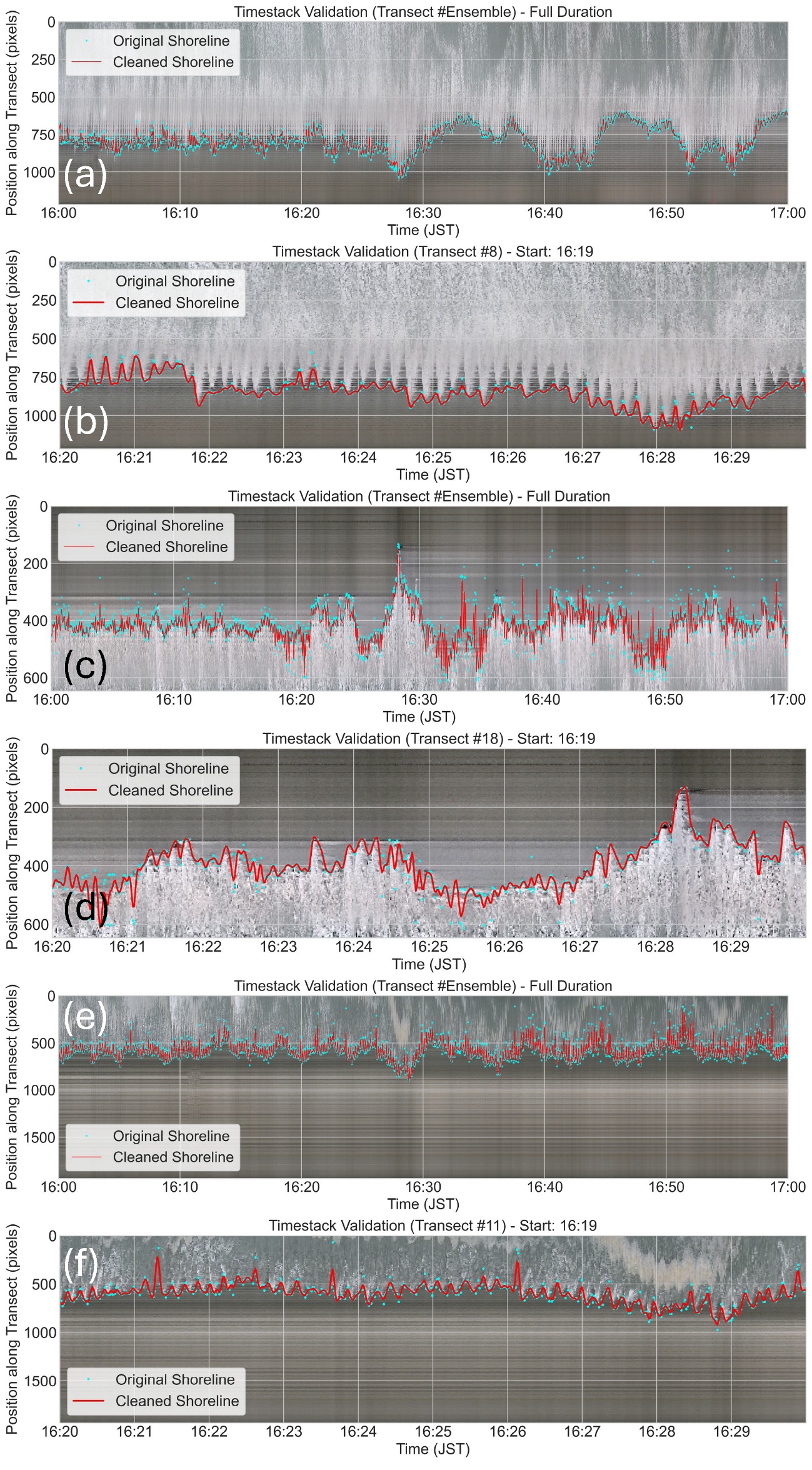
**

**Figure S6. (**Continued.)

**
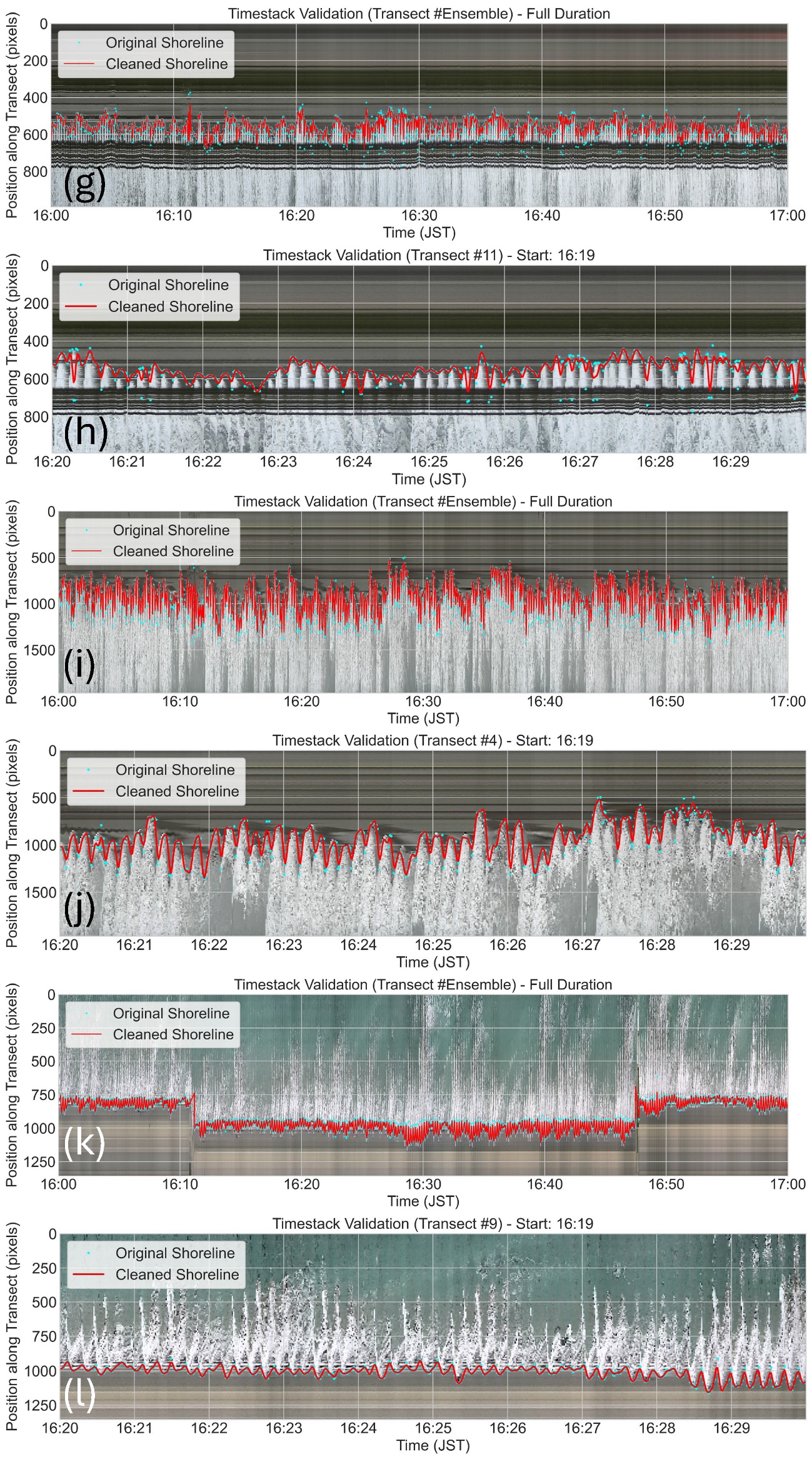
**

**Figure S6. (**Continued.)

**
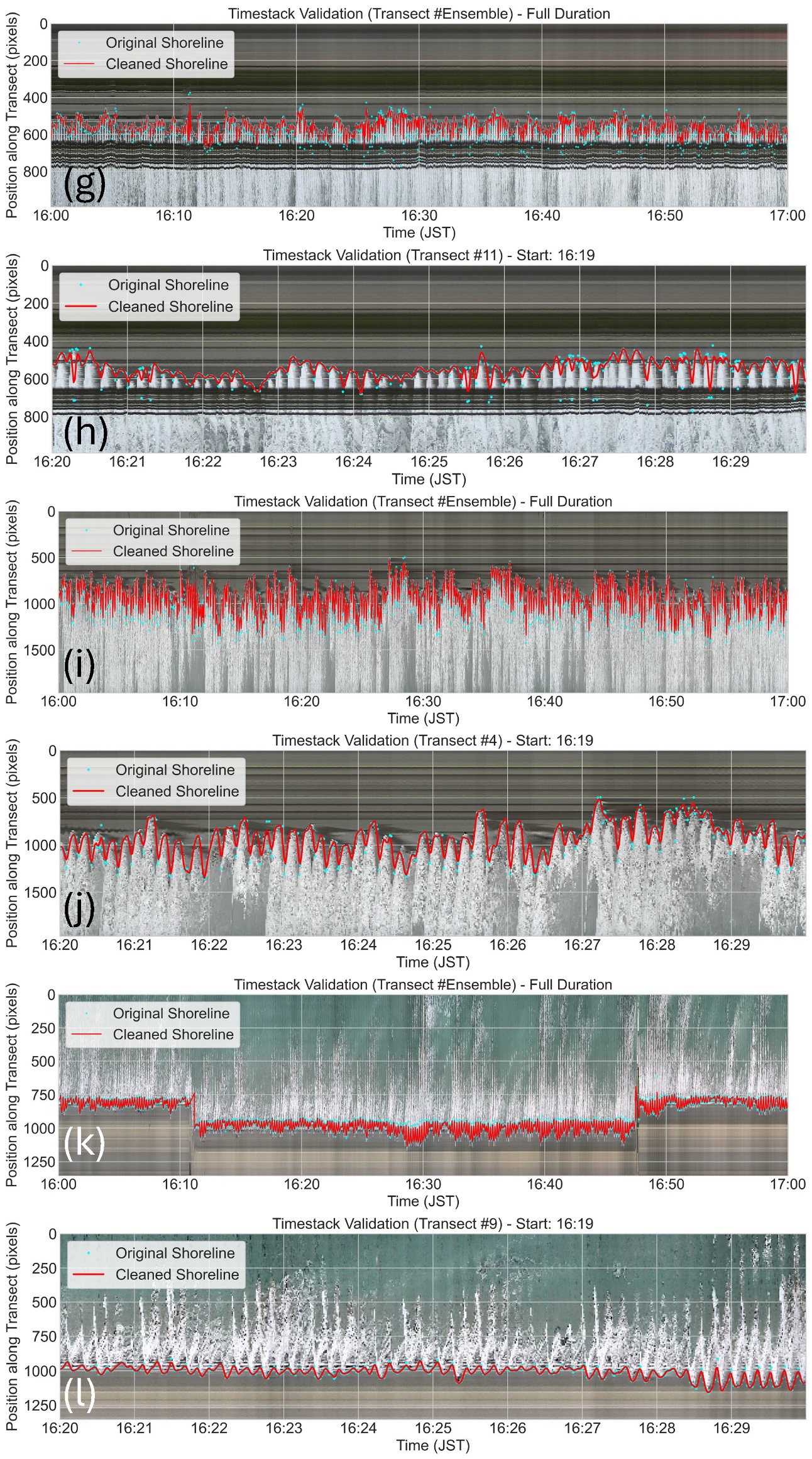

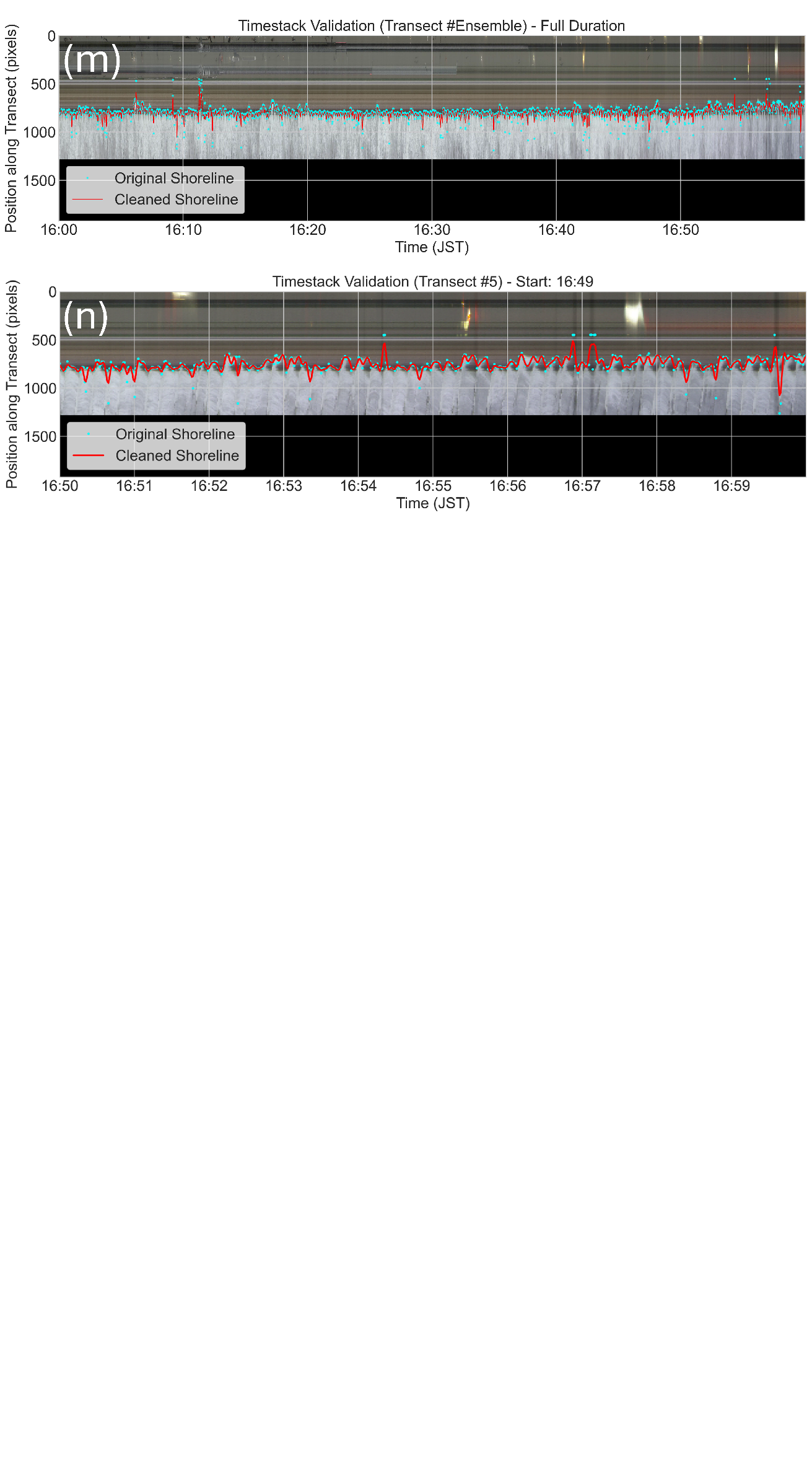
**

**Figure S6. (**Continued.)

**
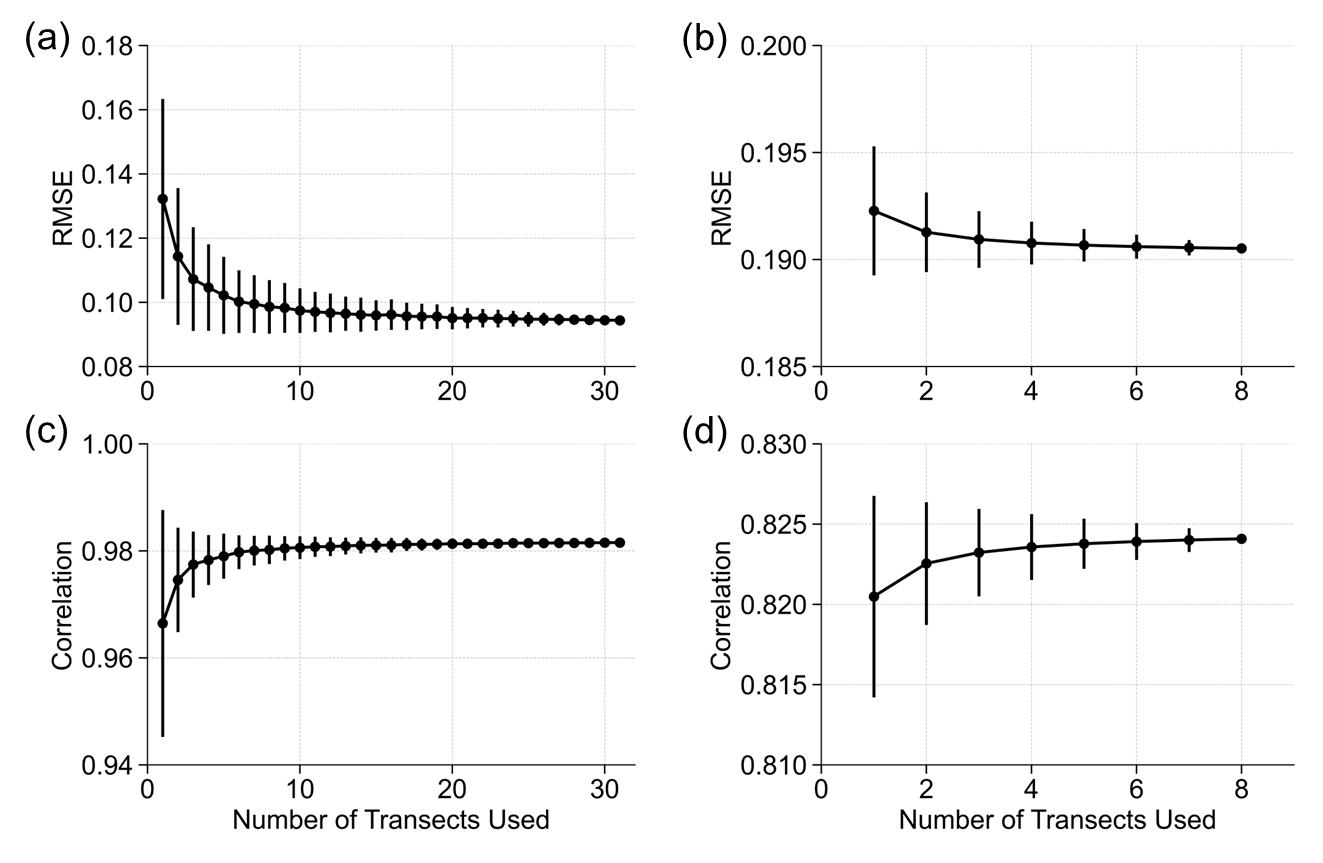
**

**Figure S7.** Performance metrics versus transect ensemble size, comparing manual and CCTV-derived runup heights—both low-pass filtered at 180 s. Panels (a) and (c) display RMSE (m) and Pearson correlation coefficient for Yokoyama, respectively; panels (b) and (d) show the same for Ekko. Error bars span the minimum–maximum range over all (or, if combinations were too many, a random subset of) transect ensembles.

**
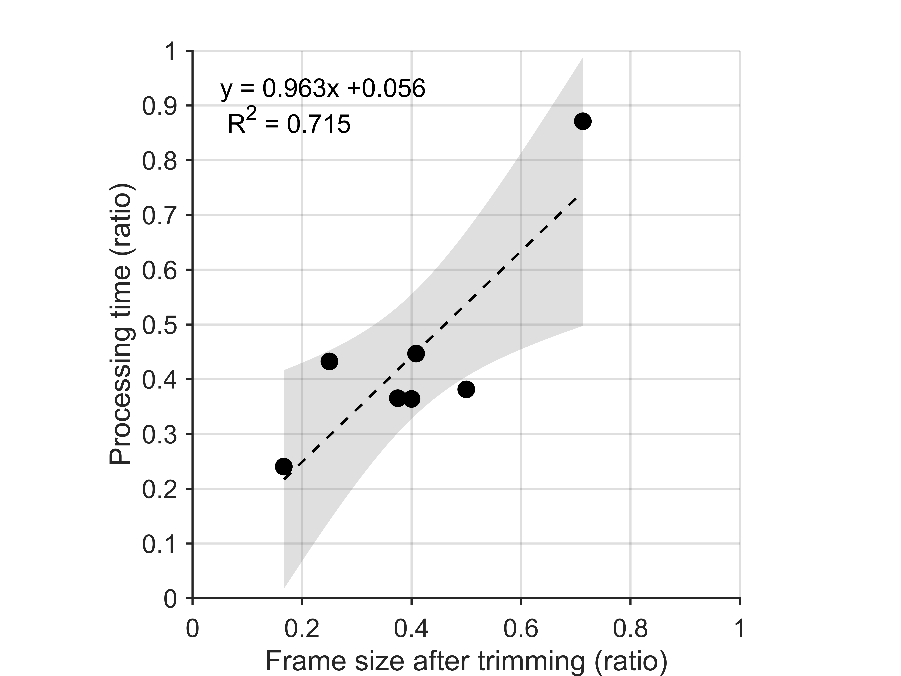
**

**Figure S8.** Relationship between trimmed‐frame size and processing time for one-hour CCTV clips from seven coastal sites. The filled black circles represent individual sites, the dashed line is the least-squares fit, and the grey band denotes its 95 % confidence interval. Processing time decreases roughly in proportion to the fraction of the original frame that is retained.


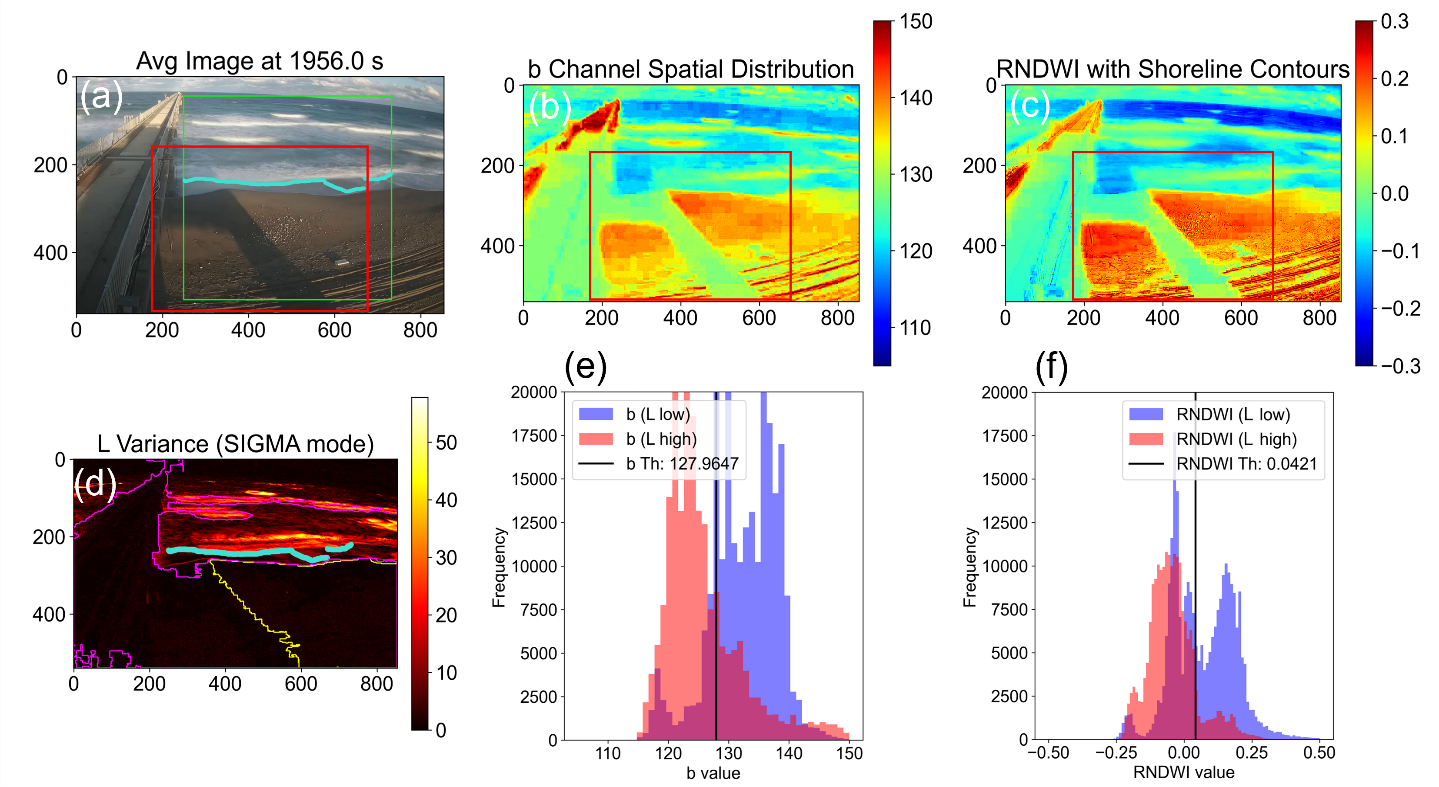


**Figure S9.** Same as Figures S1 and S2 but with localized shadows (within the red box) causing the color mask to deviate significantly from the true shoreline. In this example, the b* mask remains reasonably accurate, whereas the RNDWI-derived land/water boundary is erroneously detected along the building’s shadow.

**Text S1**. Details of the tsunami numerical simulation.

To compare the runup height time series with offshore tsunami waveforms, we utilize the fault model shown in Table S1 and corresponding numerical simulation results explored by Arikawa et al. (2024) [1]. This simulation was calibrated based on the Ministry of Land, Infrastructure, Transport and Tourism’s fault model [2] to obtain good agreement with both the offshore wave observations and water marks in wide region of the Japan Sea. The tsunami propagation calculations were performed using the JAGURS model [3], which is based on the nonlinear long wave equation, with a horizontal resolution of 0.0005 deg.

**Table S1**. The fault parameters [2] used in this study for the tsunami numerical simulation.

| Model | | Lat. | Lon. | depth (km) | Length  (km) | Width (km) | Dip  (deg.) | Strike (deg.) | Rake  (deg.) | Slip (m) |
| --- | --- | --- | --- | --- | --- | --- | --- | --- | --- | --- |
| F42 | West | 37.6983 | 137.7436 | 2.5 | 18.1 | 17.7 | 30 | 241 | 112 | 3 |
|  | East | 38.0095 | 137.8939 | 2.5 | 18.85 | 17.7 | 30 | 201 | 78 | 3 |
| F43 | West | 37.3274 | 136.6811 | 1.1 | 48.3 | 19.7 | 45 | 64 | 113 | 4.5 |
|  | East | 37.5179 | 137.1753 | 1.1 | 45.9 | 19.7 | 30 | 55 | 105 | 3 |

**References**

[1] Arikawa, T. et al. Mechanism and numerical simulation of the 2024 Noto Peninsula earthquake tsunami (in Japanese). Coastal Engineering Conference, Japan, 7 November 2024.

[2] Ministry of Land, Infrastructure, Transport and Tourism. Investigation for large earthquakes occurring in the Sea of Japan. https://www.mlit.go.jp/river/shinngikai_blog/daikibojishinchousa/ (2014). Last accessed 8 May 2025.

[3] Baba, T., Takahashi, N., Kaneda, Y., Inazawa, Y. & Kikkojin, M. Tsunami inundation modeling of the 2011 Tohoku earthquake using three-dimensional building data for Sendai, Miyagi Prefecture, Japan. In Tsunami Events and Lessons Learned: Environmental and Societal Significance (eds Kontar, Y. A., Santiago-Fandiño, V. & Takahashi, T.) 89–98 (Springer, 2014).
